# The Usefulness of a Targeted Next Generation Sequencing Gene Panel in Providing Molecular Diagnosis to Patients With a Broad Spectrum of Neurodevelopmental Disorders

**DOI:** 10.3389/fgene.2022.875182

**Published:** 2022-08-11

**Authors:** Simona Mellone, Chiara Puricelli, Denise Vurchio, Sara Ronzani, Simone Favini, Arianna Maruzzi, Cinzia Peruzzi, Amanda Papa, Alice Spano, Fabio Sirchia, Giorgia Mandrile, Alessandra Pelle, Paolo Rasmini, Fabiana Vercellino, Andrea Zonta, Ivana Rabbone, Umberto Dianzani, Maurizio Viri, Mara Giordano

**Affiliations:** ^1^ Laboratory of Genetics, Clinical Biochemistry, University Hospital Maggiore della Carità, Novara, Italy; ^2^ Department of Health Sciences, Università del Piemonte Orientale, Novara, Italy; ^3^ Child Neuropsychiatry Unit, Ospedale San Gerardo Monza-Università degli Studi di Milano Bicocca, Monza, Italy; ^4^ Department of Child Neuropsychiatry, Hospital Maggiore della Carità, Novara, Italy; ^5^ Department of Molecular Medicine, University of Pavia, Pavia, Italy; ^6^ Medical Genetics Unit, Città della Salute e della Scienza University Hospital, Torino, Italy; ^7^ Child Neuropsychiatry ASL, Vercelli, Italy; ^8^ Child Neuropsychiatry Unit, SS. Antonio e Biagio e Cesare Arrigo Hospital, Alessandria, Italy; ^9^ Division of Pediatrics, University Hospital Maggiore della Carità, Novara, Italy

**Keywords:** neurodevelopmental disorders, NGS gene panel, autism, intellectual disability, epilepsy

## Abstract

**Background:** Neurodevelopmental disorders comprise a clinically and genetically heterogeneous group of conditions that affect 2%–5% of children and represents a public health challenge due to complexity of the etiology. Only few patients with unexplained syndromic and non-syndromic NDDs receive a diagnosis through first-tier genetic tests as array-CGH and the search for *FMR1* CGG expansion. The aim of this study was to evaluate the clinical performance of a targeted next-generation sequencing (NGS) gene panel as a second-tier test in a group of undiagnosed patients with NDDs.

**Method:** A 221-gene next-generation sequencing custom panel was designed and used to analyze a cohort of 338 patients with a broad spectrum of NDDs (202 males and 136 females) including Intellectual Disability (ID), Autism Spectrum Disorders (ASD), Epilepsy, language and motor disorders.

**Results:** A molecular diagnosis was established in 71 patients (21%) and a *de novo* origin was present in 38 (64.4%) of the available trios. The diagnostic yield was significantly higher in females than in males (29.4% vs. 15.3%; *p* = 0.0019) in particular in ASD (36.8% vs. 7.6%; *p* = 0.0026) and Epilepsy (38.9% vs. 14.4% *p* = 0.001). The most involved genes were *SLC2A1*, *SCN1A, ANKRD11*, *ATP1A2*, *CACNA1A*, *FOXP1*, and *GNAS* altered in more than two patients and accounting for the 19.7% of the diagnosis.

**Conclusion:** Our findings showed that this NGS panel represents a powerful and affordable clinical tool, significantly increasing the diagnostic yield in patients with different form of NDDs in a cost- and time-effective manner without the need of large investments in data storage and bioinformatic analysis.

## Introduction

Neurodevelopmental disorders (NNDs) represent a group of heterogeneous early-onset conditions including autism spectrum disorder (ASD), intellectual disability (ID), language disorders, developmental delay (DD), and epilepsy, that globally affect 2%–5% of children (Boyle et al., 2011; American Psychiatric Association, 2013).

Different phenotypes frequently co-exist in the same patient, thus blurring the lines in the classification of individuals with these disorders. Intellectual disability (ID) is the most common NDD with a prevalence varying between 0.5 and 3% in the general population, characterized by deficits in both intellectual and adaptive functioning that first manifest during early childhood (Leonard et al., 2011). Children with intellectual disability (ID) exhibit an increased risk to present potential co-occurring developmental disorders. Epilepsy, which affects 0.7%–1.0% of the general population, represents a common comorbidity in the ID populations. Estimates of the prevalence of epilepsy in ID patients show that it increases with ID severity, varying from 16.1% to more than 50% (Robertson et al., 2015). ASD represents a broad clinical condition including deficits in social communication and interactions, repetitive behaviors, and restrictive interests (Totsika et al., 2011). As well as epilepsy ASD frequently coexists with ID: it is estimated that 40%–70% of ASD individuals suffers also from ID (Bertrand et al., 2001; Chakrabarti and Fombonne, 2001; Charman et al., 2011; Almuhtaseb et al., 2014; Jensen and Girirajan, 2017; Kazeminasab et al., 2018).

Multiple causes have been correlated to NDDs, including genetic, traumatic, and environmental factors that largely interact with each other (Van Bokhoven, 2011), and although the precise etiology remains unknown in most cases, the genetic component has been deciphered in a broad range of clinical phenotypes also thanks to ever increasingly sophisticated diagnostic tools.

The usefulness of a molecular diagnosis mainly relies on the possibility of giving information on the clinical outcome, preventing further superfluous testing, and proceeding with active monitoring of the patient. Furthermore, knowing the genetic cause of a disorder may disclose the involved biological pathway and, in some cases, suggests tailored pharmacological and behavioral treatments (Henderson et al., 2014). The current gold-standard tests for the molecular diagnosis of this heterogeneous group of conditions are mostly based on unbiased genomic approaches such as array comparative genomic hybridization (array-CGH), recommended as the first-tier genetic test (Di Gregorio et al., 2017), followed by multiple gene panels and whole-exome sequencing (WES), that are currently considered as the second-tier tests. In particular, large-scale genomic studies (WES and Whole genome sequencing, WGS) have established the pathogenic role of *de novo* and recessive variations in different forms of NDDs (Doan et al., 2019).

Despite its high performance, genetic testing is still not implemented routinely in clinical practice for patients with NDDs, mainly because of a scarcity of instrumental resources and trained specialized staff. The WES approach offers a comprehensive view of the entire mutational landscape, but it generates a huge amount of data making bioinformatics challenging especially in the diagnostic workflow. Although genome sequencing is expected to revolutionize diagnostics in the near future, gene panel sequencing remain crucial for an efficient and precise identification of pathogenic variants representing a more affordable second-tier test that, although limited to specific sets of genes, allows accurate identification of variants with greater sensitivity and lower overall cost compared to WES and WGS. In a recent meta-analysis, Stefanski et al. (2021) examined several cohorts with different forms of NDDs that had been screened either through a targeted gene panel (*N* = 73) or WES (*N* = 36). The diagnostic yield of WES was higher but the difference was not statistically significant (27.2% vs. 22.6%, *p* = 0.071) (Stefanski et al., 2021). Therefore, to date, targeted sequencing might represent an efficient approach achievable in clinical contexts, provided that well-conceived gene panels are used.

The aim of the present study was to evaluate the clinical usefulness of a 221-gene custom panel in a cohort of 338 individuals with a broad spectrum of NDDs. The presence of clinically relevant variants was detected overall in 21% of the individuals (with a significantly higher yield in females) suggesting that it might represent a powerful tool in the routine diagnostic workflow of these conditions.

## Patients and Methods

### Patients

The study retrospectively included all the 338 patients with NNDs that were referred to the Laboratory of Genetics of the University Hospital “Maggiore della Carità” in Novara (Italy) from January 2017 to May 2021 for genetic testing. All patients had been already screened by aCGH, resulting either negative or carrying a variant of uncertain significance (VUS), and were tested for *FMR1* CGG expansion to exclude Fragile X syndrome. Clinical data were obtained from the patients’ clinical charts provided by the unit or the physician that originally requested genetic testing. Information about the clinical presentation, cognitive performance scores, family history, and, when available, past diagnostic tests was used as the main source to characterize and stratify the subjects. All requests were accompanied by written informed consent obtained from the patients’ parents or legal representatives and written consent was requested for variant segregation analysis. Most patients were referred to the Genetics Unit from the Child Neuropsychiatry Unit (*N* = 264, 78.1%) and the others from the Pediatric Unit (*N* = 7, 2.1%) and the Neonatal Intensive Care Unit (*N* = 2, 0.6%) of the Hospital “Maggiore della Carità” of Novara. Moreover, since this Hospital is a tertiary reference center for specialized genetic tests and the hub center of the laboratory network in the North Eastern Piedmont quadrant for non-urgent tests, requests were also received from other hospitals in Piedmont (*N* = 37, 10.9%) and other Italian regions (*N* = 20, 5.9%).

### Gene Panel Design

The panel included 221 genes (Supplementary Table S1) found to be altered in NDDs, selected from OMIM (https://www.omim.org), medical literature (https://pubmed.ncbi.nlm.nih.gov/), the public resources SFARI (https://sfari.org/resources/sfari-gene) and Genomic England PanelApp (https://panelapp.genomicsengland.co.uk/). Both well-known disease-causing genes and a sub-group of a few candidate NDD genes were included; 106 genes are associated with autosomal dominant (AD) disorders, 30 to autosomal recessive (AR) disorders; 11 genes have both an AD and an AR inheritance, and 66 genes have been associated with X-linked (XL) diseases. For eight genes, the inheritance pattern is still controversial (Supplementary Table S1).

The genes were stratified into nine functional categories according to their main functional properties, with many involved in several functions: 1. Cell cycle regulation; 2. Cell structure and polarity; 3. Genomic stability/DNA repair; 4. Membrane polarity/electrochemical gradient; 5. Metabolic pathways; 6. Brain function/development and neuronal signaling; 7. Transcriptional/translational regulation and cell differentiation; 8. Intra- and intercellular signal transduction; 9. Vesicular trafficking. Information about their function was obtained from the scientific literature (https://pubmed.ncbi.nlm.nih.gov/), online databases including OMIM (https://www.omim.org) and GeneCards (https://www.genecards.org) (Supplementary Table S1).

### Targeted Next-Generation Sequencing

DNA was isolated from 200 μl of peripheral blood using ReliaPrepTM Blood gDNA Miniprep System (Promega, Fitchburg, WI, United States), according to the manufacturer’s protocols. The amount of DNA obtained from each sample was quantified using NanoDrop One (Thermo Fisher Scientific, Wilmington, DE, United States). To create the panel, the SureDesign software (Agilent Technologies, Santa Clara, CA, United States) was utilized and the size corresponded to 1.342 Mb. DNA libraries were prepared by SureSelectQXT Target Enrichment Kit according to the protocol for Illumina Multiplex Sequencing (Agilent Technologies, Santa Clara, CA, United States). Sequencing probes were designed to cover all coding exons ±20 bp flanking sequences from the intron-exon boundary. Sequencing baits were designed with 2X density so that each desired region was covered by at least four overlapping probes. DNA libraries were diluted to 12 pmol/l pools with 11 samples analyzed in parallel per one MiSeq sequencing run using a MiSeq sequencing reagent kit v3 150 cycles (Illumina, Inc., San Diego, CA, United States) to obtain an estimated coverage of 150X. In each sample, the estimated coverage exceeded 50 reads for over 96% of the target gene sequence.

Read alignment to the human genome reference (hg19/GRCh37), variant calling, and annotations of genetic variants were performed with the SureCall v3.5 software (Agilent Technologies, Santa Clara, CA, United States) and wANNOVAR (https://wannovar.wglab.org/) was used to annotate them. Variants showing low read depth (<20x) or poor base quality score (Phred quality score <20) were excluded.

**FIGURE 1:**
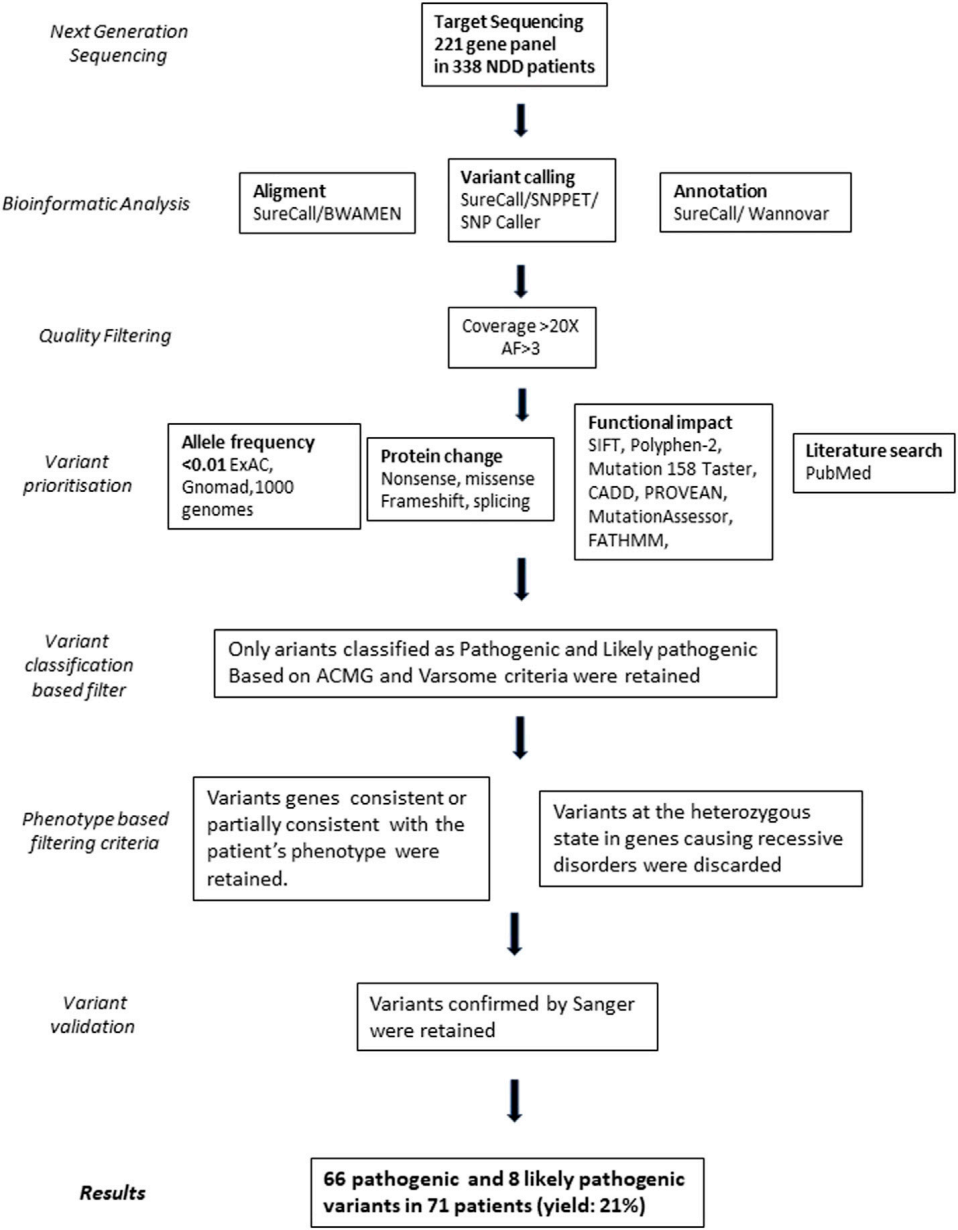
Workflow describing the main step in the identification of the selected variants. AF, Allele frequency of the variant base relative to the read number.

Variant prioritization was performed using the following bioinformatic criteria (Figure 1): 1) non-synonymous SNV or indels located in exonic or splicing regions; 2) all synonymous variants not predicted to alter the splicing mechanism were excluded; 3) allele frequency <0.01 and no homozygotes or hemizygotes if recessive in public sequence in the following databases: 1,000 Genome Project (http://browser.1000genomes.org), Exome Aggregation Consortium (ExAC) (http://exac.broadinstitute.org), Genome Aggregation Database (gnomAD) (https://gnomad.broadinstitute.org/), dbSNP (http://www.ncbi.nlm.nih.gov/projects/SNP/); 4) variant predicted to be damaging by at least four of the *in silico* predictive tools (SIFT, Polyphen-2, Mutation Taster, CADD, PROVEAN, MutationAssessor, FATHMM). Then, a literature search in PubMed (https://pubmed.ncbi.nlm.nih.gov/) was performed to investigate the role of the involved genes and to compare the phenotypes of reported patients carrying variants in the same genes (Figure 1).

Evaluation of the clinical significance of the variants was performed following the consensus guidelines published by the American College of Medical Genetics and Genomics (ACMG; https://www.acmg.net), by using the Varsome tool [https://varsome.com/, (Kopanos et al., 2019),] and by consulting the ClinVar database (https://www.ncbi.nlm.nih.gov/clinvar/). Accordingly variants were initially classified into five classes: “pathogenic,” “likely pathogenic,” of “uncertain significance,” “likely benign,” and “benign,” based on multiple lines of evidence (conservation, allele frequency in population databases, computational inferences of variant effect). In most cases, pathogenic (P) and likely pathogenic (LP) variants were definitively considered as causative after segregation analysis when parental DNA was available and after evaluating the consistency with the clinical phenotype. In essence, P/LP variants that either arose *de novo* or were inherited from an affected parent or were located within genes causing disease consistent with the patient’s phenotype were considered as the major contributors to the patient’s clinical phenotype.

### Sanger Sequencing

P and LP variants and VUS were subsequently confirmed by Sanger sequencing, which was also used to evaluate familial segregation when the parents were available. Sanger sequencing was performed using dye-terminator chemistry with the kit BigDyeTM Terminator v.1.1 Cycle Sequencing RR-100 (Applied Biosystems, Unuted States) and run on automated sequencer “SeqStudio Genetic Analyzer” (Applied Biosystems, United States). Primers were specifically designed by using the software “Primer3web” v 4.1.0 (https://primer3.ut.ee/).

### Analysis of VPS13B Through Multiplex Ligation Dependent Probe Amplification (MLPA)

Search for deletions/duplications in the *VPS13B* gene was performed by an MLPA assay using the commercial Kits SALSA MLPA Probemix P321-B3 VPS13B mix 1 and SALSA MLPA Probemix P322-C2 VPS13B mix 2 (MRC-Holland, Amsterdam, Netherlands) following the manufacturer’s instructions.

### *In-Vitro* SCN1A Minigene Splicing Assay

Fragments carrying the wild-type or mutant *SCN1A* exon 9 flanked by 338 bp of the 3′region intron 8 and 168 bp of the 5′region of intron 9 were amplified and cloned into the pSPL3 vector between the exons SD (Splice Donor) and SA (Splice acceptor) using SacI and BamHI restriction sites as previously described (Babu et al., 2021). U2O-S cells (3 × 10^5^) were seeded in a 6-well culture plate and incubated at 37°C in a 5% CO_2_ atmosphere in Dulbecco’s modified Eagle medium supplemented with 10% fetal bovine serum (Gibco-BRL, Carlsbad, CA, United States). The following day, 2 µg of wild-type or mutant vectors were transfected using Lipofectamine 2000 transfection reagent (Life Technologies, United States). cDNA was synthesized from 1 µg of RNA by the High Capacity cDNA Reverse Transcription kit (Applied Biosystems, United States), according to the manufacturer’s instructions. Using vector exon-specific primers, cDNAs produced from the mini-gene constructs were specifically PCR amplified and Sanger sequenced.

## Results

### Cohort Description

Three-hundreds-thirty-eight individuals (202 males and 136 females) with a broad spectrum of NDDs (ID, ASD, motor and/or language disorders, and dysmorphic features potentially due to a syndromic condition) were included in the study (Table 1). Epilepsy and ASD, as well as other clinical features, were present both as a comorbidity in subjects with ID and as conditions in subjects with normal cognitive function.

**TABLE 1:** Clinical characteristics of the 338 subjects and diagnostic yield.

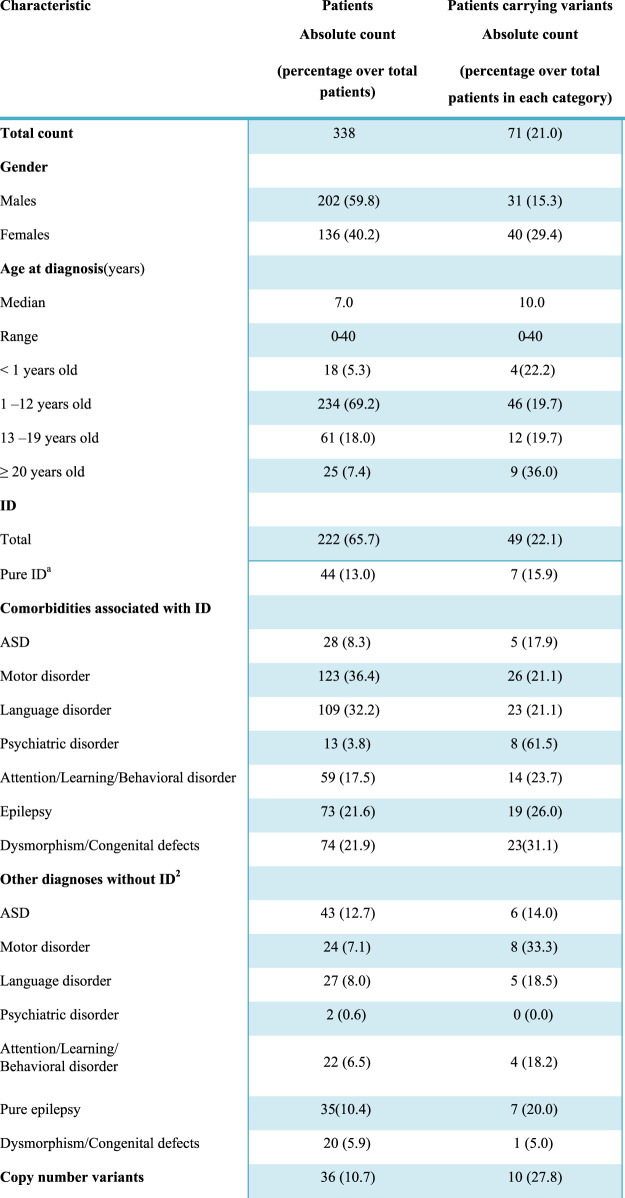

a Pure ID, refers to a diagnosis of intellectual disability in the absence of distinct comorbidities such as ASD, attention/learning/behavioral disorders, epilepsy, psychiatric disorders, and dysmorphic features. However, considering the high prevalence of motor and language impairment in subjects with ID, these two clinical features were included in the definition.

b Each additional diagnosis other than intellectual disability is considered including a potential overlap among the clinical manifestations, so that some patients may have, for instance, ASD, and epilepsy or ASD, and a learning disorder at the same time. Conversely, pure epilepsy is intended here as an isolated clinical entity, i.e. it refers to the presence of symptomatic chronic seizures in the absence of ID, motor or language disorders; ASD, attention/learning/behavioral disorders, psychiatric disorders, or dysmorphic traits.

Abbreviations: ASD, autism spectrum disorder; ID, intellectual disability; LP, likely pathogenic; P, pathogenic.

The majority of subjects were children (69.2% in the 1–12 age range) and the median age at the time of first clinical presentation was 7.0. The cohort also included 18 newborns (<12 months) and 25 young adults, so the final age ranged from 0 to 40. ID was diagnosed in 222 individuals (65.7%), based on clinical assessment and objective performance scales, such as Wechsler Preschool and Primary Scale of Intelligence-III (WPPSI-III), Griffiths Mental Development Scales (GMDS), Leiter-R scale, Vineland-II scale, or Brunet-Lézine scale. A total of 116 individuals had no ID but were affected by one or more of other conditions, in particular, ASD was present in 43 subjects, motor disorders in 24, language disorders in 27; attention deficit-hyperactivity disorder (ADHD), learning difficulties, and behavioral alterations, included in the same category were observed in 22 subjects; 35 individuals presented epilepsy without any other comorbidity; dysmorphism/congenital defects were present in 20 and psychiatric disorders in two patients (Table 1). Positive family history was referred in 32 cases and in 12 of them the heredity was compatible with an autosomal dominant/recessive disorder. It has to be considered that this might be an underestimation since it is based on the retrospective records obtained by clinicians regarding the parents and the information reported during counseling where in some cases only one accompanying family member was present and in other cases, the presence of NND signs in the relatives might not have been evaluated.

An exceeding number of males was present in the whole NND cohort, with a male-to-female ratio of 1.5. This male excess was particularly evident in ASD (with or without ID), with a prevalence in males of 25.7% compared to 13.9% of females (*p* = 0.01).

In all patients, P and LP copy number variants (CNV) were ruled out, as well as the expansion at the Fragile X locus. In 36 (10.7%) patients, aCGH had revealed a VUS that was not considered the main cause of the disorder.

### Identification of P/LP Variants

All patients were analyzed with the 221 gene NGS panel (Supplementary Table S1). A manual prioritization procedure based on expert knowledge related to the disease phenotype and gene functions detected P (*N* = 66) and LP variants (*N* = 8) in 71 patients corresponding to 21% of the whole cohort (Supplementary Table S2; Figure 1). The clinical characteristics available for patients carrying P/LP are detailed in Supplementary Table S3. Fifty-five patients were heterozygous for a variant causing AD in a single gene and one patient for two in different genes; 3 patients were compound heterozygous/homozygous for biallelic variants causing autosomal recessive disorders (#8836, #559-19, #10456); 12 patients carried variants in X-linked genes (2 males and 10 females).

To assign pathogenicity, the variants were analyzed through Varsome (https://varsome.com/), a tool for human genetic variation analysis that follows the ACMG guidelines (Kopanos et al., 2019), and the verdict for each variant was adjusted according to the variant origin and to the correlation with the clinical phenotype (PS2 and PP4 Varsome criteria, respectively). Only variants identified in patients with a clinical phenotype consistent or partially consistent with that reported in subjects with alterations in the same genes were considered. By contrast, 315 variants classified as VUS (data not shown) based on the current knowledge are not considered in the present study although they are constantly revisited.

Despite the predominance of males in our whole cohort, a significantly higher number of disease contributing variants were identified in females (29.4%) than in males (15.3%; *p* = 0.0019; Table 2).

**TABLE 2:** Diagnostic yield in males and females.

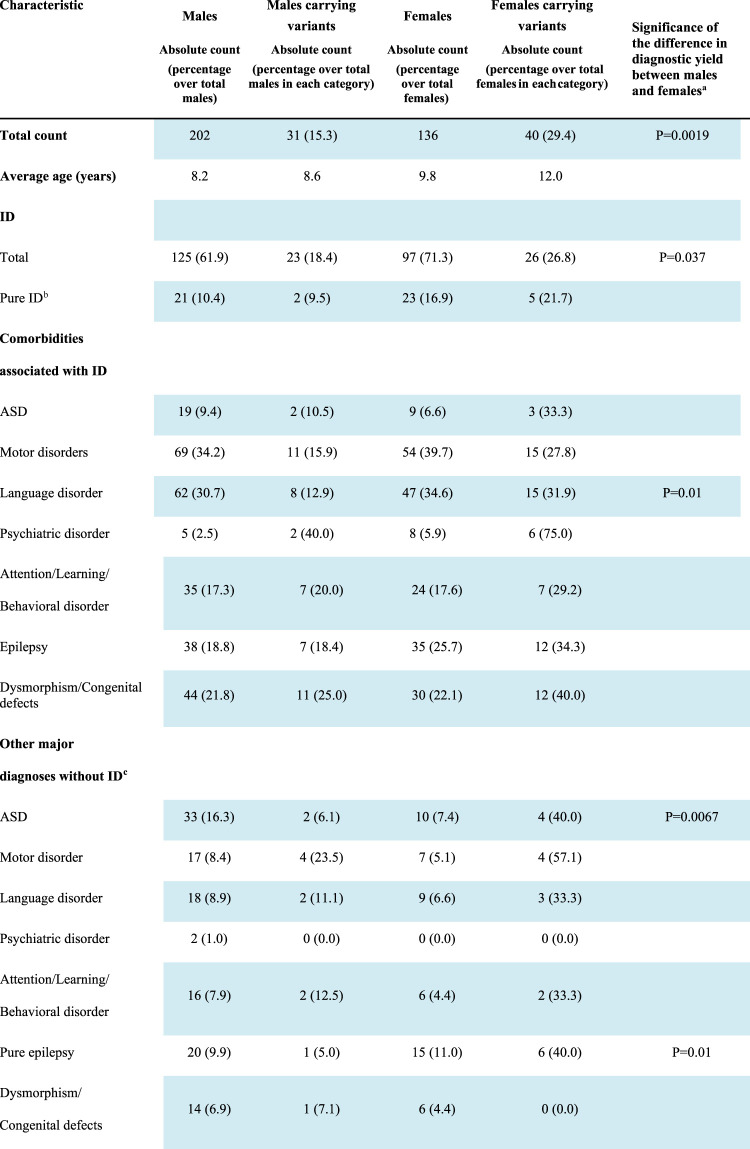

a Significance has been calculated by 2 × 2 contingency table. Only *p* value < 0.05 are reported.

b Pure ID, refers to a diagnosis of intellectual disability in the absence of distinct comorbidities such as ASD, attention/learning/behavioral disorders, epilepsy, psychiatric disorders, and dysmorphic features. However, considering the high prevalence of motor and language impairment in subjects with ID, these two clinical features were included in the definition.

c Each additional diagnosis other than intellectual disability is considered including a potential overlap among the clinical manifestations, so that some patients may have, for instance, ASD, and epilepsy or ASD, and a learning disorder at the same time. Conversely, pure epilepsy is intended here as an isolated clinical entity, i.e., it refers to the presence of symptomatic chronic seizures in the absence of ID, motor or language disorders; ASD, attention/learning/behavioral disorders, psychiatric disorders, or dysmorphic traits.

Abbreviations: ASD, autism spectrum disorder; ID, intellectual disability; LP, likely pathogenic; P, pathogenic.

Segregation analysis was performed in 60 out of the 71 cases, for whom the DNA of both parents was available, and a *de novo* origin could be established in 38 (64.4%) of the analyzed trios. Of the 13 autosomal variants involved in dominant disorders and resulting transmitted, 10 were of maternal origin and 3 of paternal origin, with a gender transmission ratio of 3.3:1 (F: M). Both males carrying X-linked P/LP variations (#10851 and #1002-19) inherited the variant from the heterozygous unaffected mother. Among the 10 females showing X-linked variants, seven carried a variant that arose *de novo* and in one case the origin could not be assessed (Table 2).

In the whole cohort, a total of 22 P/LP variants causing well-known recessive disorders were identified at the heterozygous state (data not shown). Sixteen of them were not considered correlated to the patient’s disorder owing to the lack of expected clinical features related to the involved gene (data not shown). Five patients carried monoallelic P/LP variants in *VPS13B*, mapping within the Cohen syndrome critical region on 8q22 (Kolehmainen et al., 2003), that manifest as a recessive disorder. Only one of them, #10456 (Supplementary Tables S2, 3), exhibited clinical features typical of this syndrome, including microcephaly, peculiar facies, hypotonia, non-progressive cognitive deficit, myopia, retinal dystrophy, neutropenia, and truncal obesity. Moreover, the analysis performed through the Surecall Software (Agilent) suggested that this patient might carry an intragenic *VPS13B* deletion. The specifically designed MLPA confirmed a deletion involving exons 4-7 with a minimum length of 19.46 kb. Thus the two *VPS13B* variants, that were inherited from the two heterozygous parents (Supplementary Table S3), were considered as causative of the Cohen Syndrome in this patient. The MLPA analysis was also extended to the other four patients, although they did not show any sign of Cohen syndrome, but no deletions/duplications were detected in *VPS13B*. As a consequence, their variants were not counted among the causative ones.

Among the clinically relevant variants 12.2% were variants predicted to cause a splicing defect falling within the highly conserved dinucleotide at the acceptor or donor splice site. Only one putative splicing variant, namely *SCN1A* c.695-6_695-3delTTTC in patient #10457, located at position -3 from exon 9, did not fall within the canonical dinucleotide but just upstream the acceptor splice site. Alteration of *SCN1A* (sodium voltage-gated channel alpha subunit 1) are associated with Dravet Syndrome (OMIM #607208, Supplementary Table S1), a Mendelian disorder characterized by drug-resistant epilepsy with early-onset seizures (usually during the first year of life) after apparently normal development, and usually induced by fever, at least initially (Dravet, 1978). The variant pathogenicity was evaluated following the ACMG criteria that classified it as pathogenic considering that it arose *de novo* in a patient with typical clinical features of Dravet syndrome. An *in vitro* splicing assay was set up to demonstrate the role of this novel variation. Sequencing of the PCR products from mini-genes bearing the mutant and wild type alleles revealed that c.695-6_695-3delTTTC causes aberrant splicing. The transcript originating from the wild-type construct resulted in a product of 532 bp whereas the variant construct produced a smaller transcript of 262 bp, corresponding to the size of the empty plasmid (Supplementary Figure S1). Sequence analysis of these transcripts revealed that the wild-type product results from the correct splicing of *SCNA1* exon 9 whereas the variant product results from complete skipping of exon 9 and junction of the two plasmid exons. The predicted product is a protein lacking the 90 amino acids encoded by exon 9 corresponding to a portion of the first transmembrane domain.

In the cohort there were a total of 12 individuals for whom at least one first/second degree relative with a clinical phenotype attributable to NDDs was reported, suggestive of autosomal heredity, although the phenotype was not necessarily the same observed in the index case as different diagnosis might represent variable expressivity of the same underlying genetic cause. A pathogenic variant segregating in accordance with a autosomal dominant disorder was identified in 3 of these cases (#10617,#211-20, #482-12) whereas in one case (#422-20) the variant arose *de novo*. In this latter case the patient had a sister and a first cousin (from maternal lineage) with NNDs and the mother reported four miscarriages, thus suggesting a second independent not investigated genetic alteration in this family (i.e., chromosomal balanced translocation in the mother).

### Genes Bearing Clinically Relevant Variants

Variants were identified in a total of 48 genes from the 221 genes of the panel. The most represented functional categories were those correlated to brain function and development (71%), regulation of gene expression and differentiation (58%), and cell signaling pathways (33%). Fourteen genes were mutated in more than one patient accounting for 19.7% of the cases receiving a molecular diagnosis. The most involved genes were *SLC2A1* altered in five patients, *SCNA1* in 4 patients, and *ANKRD11*, *ATP1A2*, *CACNA1A*, *FOXP1*, and *GNAS*, each altered in three patients (Supplementary Table S3).

### Complex Phenotype in a Patient With Two Pathogenic Variants

Patient # 298-19, a 12-year-old boy, carried a splicing variant in *TBX1* and a frameshift in *UBE3A* (Supplementary Tables S2, 3). Both variations were of unknown origin as they were not present in the mother, and the father’s DNA was not available. The patient was referred for suspicion of Angelman syndrome (AS), characterized by severe intellectual disability behavioral disorder and unexplained falls. He showed developmental delay (Brunet-Lézine DQ = 48), predominantly in the performance, visuo-perceptive coordination, and communication. The EEG revealed occipital paroxysms in the occipital area and MRI showed a thinning of the *corpus callosum* and reduced white matter signaling. *UBE3A* fits the AS characteristics of this patient, and it might be hypothesized that the variant arose *de novo* in the maternal germline cells, since this gene is subjected to paternal imprinting (OMIM **#** 105830). The other altered gene, *TBX1*, has been recently reported to be involved in agenesis of the *corpus callosum* and reduced white matter in haploinsufficient mice (Hiramoto et al., 2021), which were both features displayed by the patient that are not typical of AS. The complex phenotype might thus be the result of two different causative variants in different genes.

### Variants Identified in ID Patients

ID was present in 222 patients (125 males and 97 females), and in 44 of them, it was not associated with either ASD or epilepsy, behavioral disorders, or dysmorphic traits. However, considering the high prevalence of motor and/or language impairment in ID patients, these two clinical features were still considered in the definition of “pure ID.” Clinically relevant variants were found in 49 (22.1%) patients with ID, and seven of them did not present comorbidities other than motor or language difficulties. A higher proportion of females with ID (26.8%) carried causative variants in comparison to males (18.4%) although with a borderline significance (*p* = 0.037, Table 2).

### Variants Identified in Patients With Epilepsy

A total of 135 patients (76 males and 59 females) manifested epilepsy and in 73 of them it was associated with ID. Among the total, 34 (25.6%) patients carried causative variants in 23 different genes (Supplementary Table S2). Thirty-five patients manifested epilepsy without any other comorbidity and seven of them (20.0%) carried variants in six genes, namely *KCNQ2*, *NALCN*, *PAK3*, *PRRT2*, *SCN1A*, and *SLC2A1* that are among the well-known genes causing epilepsies or syndromes including epilepsy (Wang et al., 2017).

A significantly higher proportion of females with epilepsy carried pathogenic variants in comparison to males (38.9% vs. 14.4% *p* = 0.001) and the difference was also evident in isolated epilepsy (40.0% females *versus* 5.0% males; *p* = 0.01, Table 2).

### Variants Identified in Patients With ASD

ASD was present in 71 patients (52 males and 19 females) and 11 of them (15.5%) carried pathogenic variants in 11 different genes (Supplementary Table S3). All these genes were listed in SFARI (https://gene.sfari.org/), a comprehensive database including genes associated with autism, and six of them (*KANSL1, SLC9A6, CDKL5, SYNGAP, SCN1A,* and *FOXP1*) are currently classified in the first category that includes those that have been clearly implicated in ASD with high confidence.

As for epilepsy, a significantly higher proportion of females with ASD carried P/LP compared with males either with other co-morbidity (36.8% *vs*. 7.6%; *p* = 0.0026) or isolated (40.0% *vs*. 6.1%; *p* = 0.0067).

## Discussion

The clinical heterogeneity of patients suffering from NNDs and the wide spectrum of comorbidities render large genomic unbiased NGS analyses the best approach to identify the underlying genetic defects. On the one hand, WES is the gold standard method to identify pathogenic variants affecting the primary protein structure including those that are present in less frequently mutated and still undiscovered genes. On the other hand, WGS offers, at higher costs, additional benefits since it can uncover regulatory and deep intronic alterations, as well as CNV by using appropriate bioinformatics tools. However, in most clinical diagnostic centers, systematic use of WES and WGS poses the problem of the high costs due to the need of storing and analyzing huge amounts of data and co-sequencing both patients’ parents. Therefore, the creation of affordable NGS panels containing a careful selection of genes involved in the diseases of interest may be a good compromise to reach a molecular diagnosis in a cost- and time-effective manner, with the possibility of limiting the NGS analysis to the index case to detect the causal variant and then investigate the parental origin by conventional sequencing targeting of the variant. Targeted gene panels depending on the platform used, require from one third to half of the expenses needed to analyze and store the WES data of the trios in terms of reagents, bioinformatics supplies and operator costs. Thus these tests might be easily implemented in the Hospitals that already represent the tertiary reference center for specialized genetic tests.

Aiming to avoid large investments but, at the same time, to obtain adequate coverage of the selected genes (mean 150 X) with the possibility of identifying causative variants in a heterogeneous population of NND patients, we designed a panel of 221 genes that currently represent the most promising ones for our diagnostic purposes. This panel allowed us to identify a genetic defect contributing to the phenotype in 21% of the patients analyzed, which is in line with the results described in a recent meta-analysis by Stefanski et al. (2021) showing an overall yield of 22.6% for targeted gene panels sequencing, compared to 27.2% for WES. Therefore, this result can be considered a success as our cohort includes individuals with heterogeneous clinical outcomes, which limits the number of genes associated with each disorder as opposed to highly specific panels designed for homogeneous groups of patients used in several studies evaluated by the meta-analysis. Grouping patients on the basis of two main clinical phenotypes, the yield was 26.0% in epilepsy and 17.9% in ASD, in line with the data detected by the meta-analysis (24% and 17.1%, respectively).

Increased male prevalence has been repeatedly reported in several studies as well as in our NDD cohort (Table1). Epidemiologic studies report a 30%–50% excess of males in ID (Polyak et al., 2015) and, in ASD, a male-to-female ratio of 4:1 that increases to 7:1 in high-functioning autism (McLaren and Bryson, 1987). Several mechanisms might cause this bias, including sex hormones, as suggested for testosterone exposure in fetal life in ASD (Baron-Cohen et al., 2015). Possible genetic mechanisms underlying this gender seem not to involve X-linked variants, since X-linked ID is too rare to account for the 30% excess of males with this disorder and a recent burden analysis on a large group of patients with NDDs reported that X-linked causal variants were carried in similar proportions of males (6%) and females (6.9%) (Martin et al., 2021). Our panel allows good coverage of X-linked genes, since it includes 67 of the 130 X-linked genes that have been implicated in NNDs, and detected X-linked variants in two males and 10 females, with seven originated *de novo*. This enriched X-linked *de novo* variant in females is expected and might be in part explained by a higher lethality for mutations in the hemizygous male and milder phenotypes in females, in particular for those genes that escapers of X-inactivation (Tukiainen et al., 2017). In fact, it has been observed that 40% of the genes that are enriched in *de novo* mutations in females with NDDs escape X-inactivation (Turner et al., 2019), as DDX3X and *NAA10*, mutated in two patients of our cohort.

Despite the prevailing number of males present in our cohort, a significantly higher number of variants were identified in females. Global data showed relevant variants in 29.4% of females *versus* 15.3% of males (*p* = 0.0046), with even a higher bias in epilepsy and ASD (Table 2). This higher yield in females has emerged previously (Turner et al., 2019). For instance, an excess of pathogenic CNV in females has been shown in a large cohort of NDD patients with an O. R of 1.46 (*p* = 8 × 10^−8^) and an excess of deleterious SNV in females (O.R = 1.34) has been found in ASD (Jacquemont et al., 2014). These data are consistent with the “female protective model” that suggests that the clinical manifestation of NNDs in females requires a higher “mutational burden” to reach the threshold for a diagnosis.

Although family history for NNDs was referred in the 9.5% of the cases only in few of them the inheritance pattern suggested an autosomal disorder (even with reduced penetrance). The low percentage of familial cases in the whole cohort is attributable to the reduced fitness associated with NDDs and to an increased rate of *de novo* variants as recently demonstrated by a large WES study (McRae et al., 2017) reporting that these alterations account for approximately half of the genetic architecture of severe developmental disorders; in the present study *de novo* variations were identified in the 64.4% of the analyzed trios. The detection of pathogenic variants in only few familial cases might be due the limitations of the utilized gene panel that don’t cover intronic and regulatory regions and includes only most promising genes as well as to the presence of more complex pattern of inheritance in many patients. In fact the wide phenotypic variability of the NDDs likely reflects the interaction of multiple genes within an individual’s genetic background and different genetic combinations might exist among patients and affected member of the same family. The 96% of the detected VUS were missense (data not shown) and although in some families these segregated consistently with the phenotype, they remained classified as VUS. Protein disrupting and *de novo* variations are less ambiguous and in most cases classified as pathogenic by bioinformatics tools. However some of the inherited missense VUS (maybe with milder effect) might actually contribute to patients’ phenotype. Although challenging, VUS should be more extensively studied, periodically reanalyzed and much more efforts should be dedicated to decipher their functional role.

In our subjects with ID, most variants were found in genes related to transcriptional regulation, brain development, and neuronal and intracellular signaling while genes mutated in ASD were associated with the same functional categories but also with the control of cell structure and polarity. ASD is a pervasive NDD with multifactorial etiology (Walsh et al., 2008) and previous attempts to identify causative candidate genes supported the implication of synapse pathology and abnormal neural network formation as the two main pathogenic mechanisms (Rylaarsdam and Guemez-Gamboa, 2019). In line with these findings, most gene variants detected in our ASD subjects are related to the synaptic structure or signaling pathways (*CDKL5*, *DOCK8, GNAS*, *SCN1A*, *SLC9A6*, *SYNGAP1*), or transcriptional regulation with a putative role in the patterning of neural circuits (*FOXP1*) (Walsh et al., 2008; Glessner et al., 2017) (Supplementary Tables S1, 2).

In the seven patients with isolated epilepsy carrying causative variants, the genes involved play roles in synaptic signaling, cell and energy metabolism, and intracellular signal transduction. In particular, *KCNQ2, NALCN*, and *SCN1A* are responsible for ion conductance and membrane polarity, *PRRT2* codes for a protein interacting with key components of the presynaptic density, *PAK3* is involved in intracellular signal transduction pathways, and *SLC2A1* encodes GLUT1, mainly expressed in the blood-brain barrier (BBB) and working as a key transporter of glucose, the first-choice energy source in the central nervous system (CNS).

It must be underlined that *SLC2A1* was the most frequently altered gene in both comorbid and isolated epilepsy, and in the whole NDDs cohort irrespectively of the disease considered. GLUT1 deficiency syndrome has been characterized as a specific clinical entity encompassing neurodevelopmental impairment, movement disorders, and seizures, and it has been predominately associated with pathogenic *SLC2A1* variants (Graham, 2012). Early detection of this genetic alteration is clinically important, since affected subjects may benefit from ketogenic diets, which may mitigate symptoms and even prevent their progression provided that treatment is started soon when the CNS development is still not complete.

## Conclusion

This work shows the utility of a customized 221 gene panel that allowed to reach a molecular diagnosis in 21% of patients with NDDs and supports the opportunity of using a common gene panel as a second-tier test for different forms of NNDs. This approach allows to update the gene panel according to changes in the diagnostic criteria of this clinically heterogeneous group of disorders which would consequently increase the diagnostic power. Given the diagnostic yield and the potential clinical benefits, this testing should be offered to all patients with global developmental delay, intellectual disability, and/or ASD. In this scenario, WES and WGS might be reserved for the definition of complex phenotypes or familial unexplained cases, allowing a more efficient allocation of the resources.

## Data Availability

The original contributions presented in the study are included in the article/Supplementary Material, further inquiries can be directed to the corresponding author.
