# Supplementary material for: The Usefulness of a Targeted Next Generation Sequencing Gene Panel in Providing Molecular Diagnosis to Patients With a Broad Spectrum of Neurodevelopmental Disorders
Source: Front Genet. 2022 Aug 11;13:875182. doi: 10.3389/fgene.2022.875182 (PMC9403311; doi:10.3389/fgene.2022.875182)
Supplement: Supplementary file 2 [file Table1.pdf]

Supplementary Table S1. List of the 221 genes included in the NGS panel.

GENE NAME	PROTEIN NAME	CYTOGENETIC LOCATION	GENE FUNCTION	FUNCTIONAL CATEGORY*	INHERITANCE PATTERN	OMIM ENTRY
ACSL4	Acyl-CoA synthetase long chain family member 4	Xq23	Conversion of free long chain fatty acids into fatty acyl-CoA esters, key intermediates in the synthesis of complex lipids, with a preference for arachidonic acid.	5	XLD	* 300157
ACTB	Actin beta	7p22.1	Regulation of cell shape and migration, regulation of gene expression, cell division, and proliferation. Association with Baraitser-Winter syndrome 1 (BWS1, OMIM # 243310) and juvenile onset dystonia (OMIM # 607371).	2; 7	AD	* 102630
ACTL6A	Actin-like 6A	3q26.33	Required for maximal ATPase activity of BRG1 and formation of the BAF complex, involved in transcriptional activation of specific genes by antagonizing chromatin-mediated transcriptional repression.	7	N.A.	* 604958
ADNP	Activity-dependent neuroprotector homeobox	20q13.13	Homeodomain-containing zinc finger protein with transcription factor activity. Role in organogenesis and neurogenesis and transcriptional regulation to control lineage-specificity.	7	AD	* 611386
AFF2	AF4/FMR2 family, member 2	Xq28	RNA-binding protein that may be involved in alternative splicing regulation through an interaction with G quartet RNA structure.	7	XLR	* 300806
AGO1	Argonaute RISC Component 1	1p34.3	RNA interference and signaling through interaction with miRNA and siRNA	7	N.A.	* 606228
AGTR2	Angiotensin II receptor type 2	Xq23	Role in fetal development and programmed cell death through mitogen-activated protein kinases. Largely expressed in the cerebellum.	8	XLR	* 300034
AKT3	AKT Serine/ Threonine Kinase 3	1q43-q44	Role in multiple signaling pathways, particularly related to vascular, limb and brain development.	6; 8	AD	* 611223

ALDH7A1	Aldehyde dehydrogenase 7 family member A1	5q23.2	Aldehyde dehydrogenase involved in lysine catabolism. Linked to pyridoxine-dependent epilepsy.	5	AR	* 107323
ANKRD11	Ankyrin repeat-containing cofactor 1	16q24.3	Transcriptional regulator (inhibition of ligand-dependent transactivation through recruitment of H deacetylases)	7	AD	* 611192
AP1S2	Adaptor-related protein complex 1 sigma 2 subunit	Xp22	Subunit of the heterotetrameric adaptor protein-1 complex in the cytosolic side vesicles of the Golgi complex. Role in chlatrin recruitment and recognition of sorting signals from transmembrane receptors.	9	XLR	* 300629
ARHGEF6	Rho guanine nucleotide exchange factor 6	Xq26.3	Cytoplasmic protein activating the Ras-like family of Rho proteins by exchanging bound GDP for GTP. Role in the organization of the cytoskeleton, cell shape, and motility, integrin-mediated signaling leading to activation of GTPases, direction of neurite outgrowth.	2; 6; 8	XL	* 300267
ARID1A	AT-rich interaction domain 1A	1P36.11	Part of a chromatin-remodeling complex favoring transcriptional activation.	7	AD	* 603024
ARID1B	AT-rich interaction domain 1B	6q25.3	Chromatin remodeling.	7	AD	* 614556
ARX	Aristaless related homeobox	Xp21.3	Homeodomain transcription factor with a crucial role in cerebral development and patterning.	7	XL	* 300382
ASH1L	ASH-like histone lysine methyltransferase	1q22	H3K36 methylation and transcriptional regulation, nucleotide excision repair.	7	AD	* 607999
ASPM	Abnormal spindle-like microcephaly-associated	1q31.3	Essential role in mitotic spindle function in embryonic neuroblasts determinant of cerebral cortical size.	2; 6	AR	* 605481
ATP1A2	ATPase Na^+/K^+ transporting subunit alpha 2	1q23.2	Maintainance of the Na+/K+ electrochemical gradient across the plasma membrane	9	AD	* 610528

ATRX	ATRX chromatin remodeler	Xq21.1	Helicase involved in nucleotide excision repair, chromatin remodeling and transcriptional regulation, chromosomal segregation during mitosis through binding to H1P of the pericentrometric heterochromatin.	7	XLD/XLR	* 300032
AUTS2	Activator of transcription and developmental regulator	7q11.22	Part of the PRC1-AUTS2 Polycomb repressive complex involved in transcriptional activation in contrast to the canonical role of PRC1 in gene repression. Epigenetic modulation in the context of neurodevelopment and neuronal function.	6; 7	AD	* 607270
BRWD3	Bromodomain- and WD repeat-containing protein 3	Xq13	JAK/STAT signaling.	8	XLR	* 300553
C10orf11	Leucine-rich melanocyte differentiation-associated protein	10q22.2-q22.3	Melanocyte differentiation and function. Association with oculocutaneous albinism type VII (OMIM # 615179).	7	AR	* 614537
CACNA1A	Calcium voltage-gated channel subunit alpha1 A	19p13	Voltage-dependent calcium channels (this gene encodes the alpha-1A subunit, which is predominantly expressed in neuronal tissue) involved in neurotransmitter release, gene expression, and cerebellar programming and development.	6; 8	AD	* 601011
CACNG2	Calcium voltage-gated channel auxiliary subunit gamma 2	22q12.3	Brain-specific transmembrane AMPA receptor regulatory protein involved in modulation of AMPA receptor trafficking and kinetics, shaping of synaptic responses. Subunit of neuronal voltage-gated calcium channels.	6	AD	* 602911
CAMK4	Calcium-calmodulin-dependent protein kinase	5q22.1	Role in calcium-mediated activity and dynamics, particularly in the brain (neuronal transmission, synaptic plasticity, and neuronal gene expression)	6; 8	N.A.	* 114080
CASK	Calcium/calmodulin-dependent serine protein kinase	Xp11.4	Ca^{++}/calmodulin-dependent serine protein kinase, member of the membrane-associated guanylate kinase (MAGUK) family of scaffolding proteins. Role in synaptic vesicle exocytosis, formation and regulation of presynaptic and postsynaptic sites, signal transduction, cerebrocortical development through transcriptional regulation.	6; 8; 9	XLD	* 300172
CC2D1A	Coiled-coil and C2 domain-containing 1A	19p13.12	Endocytosis regulation and endosomal trafficking.	9	AR	* 610055
CCDC88C	Coiled-coil domain-containing protein 88C	14q32.11 -q32.12	Negative regulator of Wnt pathway by promoting beta-catenin degradation.	8	AD/AR	* 611204

CDH15	Cadherin 15	16q24.3	Glycoprotein mediating Ca^{2+}-dependent intercellular adhesion by homophilic interactions.	8	AD	* 114019
CDK13	Cyclin-dependent kinase13	7p14.1	DNA damage response gene regulation, genomic stability.	3	AD	* 603309
CDK5R1	Cyclin dependent kinase 5 regulatory subunit 1	17q11.2	Cofactor of CDK5 (especially in brain) with a role in cell cycle regulation, development of central nervous system, and neuronal-glial interaction in radial migration.	1; 6	AD	* 603460
CDKL3	Cyclin-dependent kinase-like 3	5q31.1	Homology to both MAP-kinases and cyclin-dependent kinases. Role in dendrite morphogenesis, positive regulation of dendrite extension and negative regulation of axonal extension.	1; 2; 8	AD	* 608459
CDKL5	Cyclin-dependent kinase-like 5	Xp22.13	Kinase with tissue-specific alternative splicing (expression almost exclusively in cerebellum, cortex, hippocampus, and olfactory bulb). Role in the control of nuclear speckle morphology.	2	XLD	* 300203
CEP83	Centrosomal protein 83	12q22	Docking of ciliary vesicles derived from the Golgi complex to the mother centriole for primary cilium assembly. Key component of the centriolar distal appendages. Role in regulating the mechanical features of neural progenitor cells and size and configuration of the cerebral cortex.	2; 6; 9	AR	* 615847
CHD1L	Chromodomain helicase DNA binding protein 1 like	1q21.1	Chromatin remodeling and relaxatiom in response to DNA strand breaks through interaction with poly(ADP-ribose) (PAR).	7	XLR	* 613039
CHD2	Chromodomain helicase DNA binding protein 2	15q26.1	Chromatin remodeling, neural circuit and long-term memory development.	6; 7	AD	* 602119
CHD7	Chromodomain helicase DNA binding protein 7	8q12.2	Neural crest cell reprogramming, transcriptional regulator in the nucleoplasm, ribosomal biogenesis in the nucleolus.	7	AD	* 608892
CHD8	Chromodomain helicase DNA binding protein 8	14q11.2	Nuclear protein involved in the negative regulation of Wnt signaling through binding to beta-catenin. Role in chromatin insulation and remodeling.	7; 8	AD	* 610528

CIC	Capicua transcriptional repressor	19q13.2	Transcriptional repressor. Role in cerebellar development and hypoxia tolerance.	7	AD	* 612082
CNTNAP2	Contactin-associated protein-like 2	7q35-q36	Neuronal transmembrane protein of the neurexin superfamily involved in neural-glia interactions and clustering of potassium channels in myelinated axons. Enriched in circuits involved in higher cortical functions, including language.	6	AD/AR	* 604569
COL4A3BP	Collagen type IV alpha 3 binding protein	5q13.3	Hydrophilic cytoplasmic protein with a phosphatidyl-inositol-4-monophosphate-binding domain and a lipid transfer-catalyzing domain for intracellular trafficking of ceramide in a nonvesicular manner.	5	AD	* 604677
CRADD	CASP2 and RIPK1 domain containing adaptor with death domain	12q22	Adaptor in DNA damage-induced apoptosis and inflammation (PIDDosome).	1	AR	* 603454
CRBN	Cerebron	3p26.2	ATP-dependent protease involved in selective degradation of short-lived polypeptides through its ubiquitin-ligase activity, regulation of mitochondrial replication and transcription, assembly and surface expression of functional BK (Ca) channels by direct interaction with the cytosolic C-terminus of its alpha-subunit.	7; 8	AR	* 609262
CREBBP	CREB-binding protein	16p13.3	Lysine acetyl transferase involved in histone acetylations, transcriptional regulation (together with EP300) and genomic stability (DNA repair mechanisms).	3; 7	AD	* 600140
CTNNB1	Catenin beta-1	3p22.11	Adherens junction protein involved in establishment and maintenance of epithelial layers and cell adhesion. Role in intercellular signaling, anchoring of the actin cytoskeleton, regulation of normal cell growth and behavior. Transmission of the 'contact inhibition' signal to prevent further cell division. Role in dendritic morphogenesis and arborization.	2; 6; 8	AD	* 116806
CTNND2	Catenin delta 2	5p15.2	Armadillo-like protein (bearing a series of armadillo repeats) involved in regulation of synapse-specific transcription of Rapsyn at the neuromuscular junction, neural development and cell motility.	6	AD	* 604275
CUL4B	Cullin 4B	Xq24	Scaffold protein of the cullin 4B-RING ubiquitin ligase (E3) complex involved in regulation of cellular protein degradation, nucleotide excision repair and DNA damage response, regulation of histone methylation.	3; 7	XLR	* 300304
DDX3X	DEAD-box RNA helicase3	Xp11.4	DEAD-box RNA helicase involved in cell cycle control, tumorigenesis, apoptosis, regulation of Wnt-β-catenin pathway, translation, and inflammasome assembly.	1; 8	XLD/XLR	* 300160

DEAF1	DEAF1 transcription factor	11p15.5	Transcriptional activator and repressor with a possible role in early neurodevelopment. Association with Vulto-van Silfout-de Vries s yndrome (VSVS, OMIM # 615828)	7	AD/AR	* 602635
DHCR7	7-dehydrocholesterol reductase	11q13.4	Cholesterol biosynthesis and embryonic signaling through the hedgehog pathway	5; 8	AR	* 602858
DIP2A	Disco interacting protein 2 homolog A	21q22.3	Axon pathfinding and patterning. Regulation of spine morphogenesis via acetylation of cortactin.	2; 6	AD	* 607711
DLG2	Discs large MAGUK scaffold protein 2	11q14.1	Regulation of the distribution and clustering of voltage-gated and ligand-gated ion channels on the neuronal surface.	6	N.A.	* 603583
DLG3	Discs large MAGUK scaffold protein 3	Xq13.1	Synapse-associated protein of the membrane-associated guanylate kinase (MAGUK) protein family involved in regulation of epithelial polarity, organization of receptors, particularly NMDA receptors (role in long-term potentiation and synaptic plasticity), and downstream signaling pathways within the synapse.	2; 6; 8	XLR	* 300189
DOCK8	Dedicator of cytokinesis 8	9p24.3	Organization of filamentous actin	2	AD	* 611432
DSCAM	Down Syndrome cell adhesion molecule	21q22.2	Cell adhesion molecule of the immunoglobulin superfamily expressed mainly in the brain with different neuron-specific isoforms. Role in axonal pathfinding, neuronal wiring, self-recognition and heteroneuronal self-avoidance.	2; 6	AD	* 602523
DYNC1H1	Dynein cytoplasmic 1 heavy chain 1	14q32.31	Subunit of the cytoplasmic dynein complex, microtubule-activated ATPases converting chemical energy into mechanical energy. Role in intracellular motility, retrograde axonal transport, protein sorting, and redistribution of organelles, mitotic spindle formation and neuronal migration.	2; 6	AD	* 600112
DYRK1A	Dual-specificity tyrosine phosphorylation-regulated kinase 1A	21q22.13	Role in several cellular processes generally resulting in increased cellular proliferation and reduced apoptosis. Role in microtubule stability in growing axons. Phosphorylation of alpha-synuclein to facilitate its intracytoplasmic aggregation (possible association with alpha-synucleinopathies). Phosphorylation of tau, reducing its function in microtubule assembly (possible role in Down syndrome-associated Alzheimer disease).	1; 2; 6	AD	* 600855

EHMT1	Euchromatic histone lysine methyltransferase 1	9q34.3	Methyltransferase with transcriptional repression activity through methylation of lysine-9 of histone H3. Potential role in frontal cortex aging and in age-related behavioral deterioration.	7	AD	* 607001
EP300	E1A-binding protein	22q13.2	Histone acetyltransferase with a role in transcriptional regulation and DNA repair.	3; 7	AD	* 602700
EPB41L1	Erythrocyte membrane protein band 4.1 like 1	20q11.23	Interactions between the erythrocyte cytoskeleton and the overlying plasma membrane, regulation of AMPA receptor expression at the synaptic membrane.	2; 6	AD	* 602879
FBXO11	F-box only protein 11	2p16.3	Methyltransferase activity. transcriptional regulation, silencing of p53 and of the mismatch repair gene MSH6.	3; 7	AD	* 607871
FGD1	FYVE, RhoGEF and PH domain containing 1	Xp11.21	Guanine nucleotide exchange factor (GEF) involved in regulation of subcortical actin cytoskeleton and Golgi complex, excretory cell morphogenesis and cellular organization, regulation of cell shape together with cortactin and mammalian actin-binding protein-1 mAbp1), invadopodia biogenesis and extracellular matrix degradation in cancer.	2	XLR	* 300546
FMR1	FMR1 antisense RNA 1	Xq27.3	Polysome-associated protein involved in mRNA trafficking and translational control, regulation of microtubule dynamics, and translation-independent modulation of presynaptic action potential. Possible role in the DNA damage response. The expansion of a trinucleotide repeat (CGG) in the 5' UTR to 55-230 copies is associated with fragile X syndrome (OMIM # 300624), fragile X tremor/ataxia syndrome (OMIM # 300623), and premature ovarian failure (POF1, OMIM # 311360).	3; 6; 7	XLR	* 300805
FOXG1	Forkhead box G1B	14q12	Transcriptional repressor involved in control of neuronal migration and formation of cortico-cortical callosal projections.	6; 7	AD	* 164874
FOXP1	Forkhead box P1	3p13	Transcriptional repressor involved in monocyte differentiation and macrophage function, pooling and columnar organization of spinal motor neurons.	6; 7	AD	* 605515
FTSJ1	FTSJ RNA 2-prime-O-methyltransferase 1	Xp11.23	Nucleolar methyltransferase involved in ribosomal stability.	7	XLR	* 300499

GABRA1	Gamma-aminobutyrric acid receptor alpha-1	5q34	Pentameric ligand-gated chloride channel acting as the receptor of the brain major inhibitory neurotransmitter, GABA, and site of action of a number of important pharmacologic agents including barbiturates, benzodiazepines, and ethanol. Association with developmental and epileptic encephalopathy (OMIM # 615744) and susceptibility to childhood absence and juvenile myoclonic epilepsy (OMIM # 611136).	6	AD	* 602523
GABRB3	Gamma-aminobutyrric acid receptor beta-3	15q12	Member of the GABA-A receptor gene family of heteromeric pentameric ligand-gated ion channels involved in GABA-ergic (inhibitory) neurotransmission Association with childhood absence and juvenile myoclonic epilepsy (OMIM # 611136) and with developmental and epileptic encephalopathy (OMIM # 615744).	6	AD	* 137192
GATAD2B	GATA zinc finger domain containing 2B	1q21.3	Regulation of transcription by linking 2 independent chromatin-regulating activities: histone deacetylation and ATP-dependent nucleosome remodeling.	7	AD	* 614998
GDI1	GDP dissociation inhibitor 1	Xq28	Vesicular trafficking, it recycles Rab proteins and keeps a reservoir of available Rab proteins in the cytosol by extracting them from membranes and by solubilizing them.	9	XLD	* 300104
GLDC	Glycine decarboxylase	9p24.1	Pyridoxal phosphate-dependent glycine decarboxylase, P protein part of an enzymatic complex (P, L, H, T) involved in glycine cleavage in mitochondria	5	AD/AR	* 238300
GNAS	GNAS Complex Locus	20q13.32	Imprinted complex locus encoding 4 transcripts (Gs-alpha, XLAS, NESP55, and the A/B transcript, as well as an antisense GNAS transcript, GNASAS). Gs-alpha is expressed biallelically in nearly all tissues. Other transcripts produced by GNAS are expressed exclusively from either the paternal or the maternal GNAS allele. Association with pseudohypoparathyroidism (OMIM # 612463) and progressive osseous heteroplasia (OMIM # 166350).	8	AD	* 139320
GNB1	Guanine nucleotide-binding protein beta-1	1p36.33	Subunit of G proteins, involved in extracellular signal transduction.	8	AD	* 139380
GPD2	Glycerol-3-phosphate dehydrogenase 2	2q24.1	Glycerol-phosphate shuttle: conversion of G-3-P into dihydroxyacetone phosphate (DHAP) with concomitant reduction of FAD to trasnfer reducing equivalents into mitochondria and reoxidize NADH.	5	AD	* 138430

GRIA3	Glutamate ionotropic receptor AMPA type subunit 3	Xq25	Glutamate receptor involved in synaptic plasticity, learning and memory.	6	XLR	* 305915
GRIK2	Glutamate ionotropic receptor kainate type subunit 2	6q16.3	Short- and long-term plasticity in hippocampal mossy fibers and modulators of synaptic strenght.	6	AR	* 138244
GRIN1	Glutamate ionotropic receptor N-methyl-D-aspartate (NMDA) type subunit 1	9q34.3	Synaptic function, dendritic arborization, synaptic plasticity, neuronal survival, potential for excitotoxicity.	6	AD/AR	* 138249
GRIN2A	Glutamate ionotropic receptor NMDA type subunit 2A	16p13.2	Neuronal excitability, synaptic plasticity and neuronal morphogenesis. Role in memory formation signaling cascades.	6	AD	* 138253
GRIN2B	Glutamate-activated NMDA receptor subunit 2B	12p13.1	Neuronal excitability, synaptic plasticity and neuronal morphogenesis.	6	AD	* 138252
HCFC1	Host cell factor C1	Xq28	Transcriptional regulation, regulation of embryonic neural development.	7	XLR	* 300019
HDAC2	Histone deacetylase 2	6q21	Zinc-finger transcription factor involved in positive and negative transcriptional regulation of consensus DNA elements at gene promoters through histone deacetylation. Modulation of synaptic plasticity and long-lasting changes of neural circuits, involvement in learning and memory.	6; 7	N.A.	* 605164
HDAC4	Histone deacetylase 4	2q37.3	Class II histone deacetylase targeting all 4 histones, chromatin remodeling.	7	XLR	* 605314
HNMT	Histamine N-methyltransferase	2q22.1	N(tau)-methylation of histamine using S-adenosyl-L-methionine as the methyl donor and control of its neurotransmitter activity in the brain.	6	AR	* 605238
HPRT1	Hypoxanthine-guanine phosphoribosyltransferase 1	Xq26.2	Generation of purine nucleotides through the purine salvage pathway (conversion of hypoxanthine to inosine monophosphate and guanine to guanosine monophosphate via transfer of the 5-phosphoribosyl group from 5-phosphoribosyl 1-pyrophosphate). Association with Lesch-Nyhan syndrome (OMIM # 300322).	5	XLR	* 308000

HUWE1	HECT, UBA, and WWE domains-containing protein 1	Xp11.2	Ubiquitin ligase involved in several cellular processes, including neurogenesis, neural proliferation and synaptogenesis. Association with Turner type X-linked mental retardation (OMIM # 309590).	6; 8	XL	* 300697
IDS	Iduronate 2-sulfatase	Xq28	Lysosomal degradation of the glycosaminoglycans heparan sulfate and dermatan sulfate. Association with mucopolysaccharidosis type II (Hunter syndrome, OMIM # 309900).	5	XLR	* 300823
IL1RAPL1	Interleukin 1 receptor accessory protein like 1	Xp21.3-p21.2	High levels of expression in brain neurons. Regulation of neurite outgrowth and exocytosis via its interaction with neuronal calcium sensors and the downregulation of calcium channels, regulation of synaptic formation and modulation of synaptic transmission.	6	XLR	* 300206
IQSEC2	IQ motif and SEC7 domain-containing protein 2	Xp11.22	GDP-GTP exchange factor involved in neuronal development and synaptic plasticity (cytoskeletal organization, dendritic spine formation and morphology, and excitatory synaptic organization).	6; 8	XLD	* 300522
JARID2	JUMONJI, AT-rich interactive domain 2	6p22.3	Regulator of histone methyltransferase complexes, transcriptional repression by binding to the Polycomb repressive complex 2 (PRC2), neural tube formation.	7	AD	* 601594
KANK1	KN motif and ankyrin repeat domains 1	9p24.3	Regulation of actin polymerization and inhibition of cell migration, regulation of Rho GTPases in podocytes). Possible association with nephrotic syndrome). Maternally imprinted gene.	2	AD	* 607704
KANSL1	KAT8 regulatory NSL complex subunit 1	17q21.31	Nuclear protein belonging to the histone acetyltransferase complex with a role in chromatin modification through acetylation of histone 4.	7	AD	* 612452
KAT6B	Lysine acetyltransferase 6B	10q22.2	Histone acetyltransferase and transcriptional regulator.	7	AD	* 605880
KATNAL1	Katanin catalytic subunit A1 like 1	13q12.3	Microtubule organization. Essential for spermiogenesis and male fertility.	2	AD	* 614764
KCNAB1	Potassium voltage-gated channel subfamily A member regulatory beta subunit 1	3q25.31	Part of the Shaker-related family of potassium channels involved in determination of the electrical properties of excitable cells.	4; 6	AD	* 601141

KCNQ2	Voltage-gated potassium channel, subfamily Q, member 2	20q13.33	Voltage-gated potassium channel involved in neuronal excitability and synaptic response.	4; 6	AD	* 602235
KCNT1	Potassium channel, subfamily T, member 1	9q34.3	Na-activated K channel contributing to neuronal hyperpolarization following repetitive firing.<br>Interaction with FMRP (possible link between changes in protein translation and neuronal firing).	6	AD	* 608167
KDM5B	Lysine demethylase 5B	11q13.2	H3K4 demethylation --> gene repression	6; 7	AR	* 605393
KDM5C	Lysine demethylase 5C	Xp11.22	H3K4me3 and H3K4me2 demethylase, transcriptional repressor through the RE-1-silencing transcription factor (REST) complex. Role in brain function.	6; 7	XLR	* 314690
KDM6A	Lysine demethylase 6A	Xp11.3	Removal of histone H3 lys27 repressive trimethylation (H3K27me3). Role in anteroposterior embryonic development.	7	XLD	* 300128
KIRREL3	Kirre-like nephrin family adhesion molecule 3	11q24.2	Part of the immunoglobulin superfamily proteins interacting with the C-terminus of podocin. Role in neuronal migration, axonal fasciculation, and synapse formation and scaffolding.	2; 6	AD	* 607761
KMT2A	Lysine-specific methyltransferase 2A	11q23.3	H3K4 methylation, chromatin remodeling and positive regulation of gene expression, neurogenesis in postnatal brain, maintainance of regionally distinct developmental<br>programs in the forebrain through epigenetic mechanisms,<br>DNA damage response and S-phase checkpoint.	1; 6; 7	AD	* 159555
KMT2D	Lysine-specific methyltransferase 2D	12q13.12	Chromatin remodeling through H3K4me3 methylation.	7	AD	* 602113
KMT5B	Lysine methyltransferase 5B	11q13.2	Nucleosomal histone H4 trimethylation.	7	AD	* 610881
L1CAM	L1 cell adhesion molecule	Xq28	Integral membrane glycoprotein belonging to the immunoglobulin superfamily cell adhesion molecules (CAMs), mediation of cell-to-cell adhesion at the cell surface. Expressed primarily in the nervous system, acting as a neural recognition molecule. Association with agenesis of the	6; 8	XLR	* 308840

			corpus callosum, hydrocephalus and CRASH and MASA syndromes (hereditary spastic paraplegias, OMIM # 303350).			
LAMA1	Laminin alpha-1	18p11.31	Basement membrane protein	2	AR	* 150320
LARGE1	Acetylglucosaminyltransferase-like protein	22q12.3	Member of the N-acetylglucosaminyltransferase gene family involved in glycosylation of alpha-dystroglycan and synthesis of glycoprotein and glycosphingolipid sugar chains. Association with congenital muscular dystrophy-dystroglycanopathy with mental retardation type A6 (OMIM # 613154) and B6 (OMIM # 608840).	5	AR	* 603590
LINS1	LINES homolog 1	15q26.3	Modulator of WnT signaling pathway.	8	AR	* 610350
MAGEL2	MAGE-like 2	15q11.2	Ubiquitin ligase enhancer required for endosomal protein recycling. Role in brain development, hypothalamic function, possible role in circadian rythm control and age at menarche determination with a parent-of-origing imprinting effect. Association with Schaaf-Yang syndrome (OMIM # 615547).	6; 9	AD	* 605283
MAGT1	Magnesium transporter 1	Xq13.1-q13.2	Magnesium transport. Association with X-linked immunodeficiency with magnesium defect and congenital disorder of glycosylation.	4	XLR	* 300715
MAP3K7	Mitogen-activated protein kinase kinase kinase 7	6q15	Kinase-mediated cascade signaling pathways, immune response, TGF-β signaling.	8	AD	* 602614
MBD5	Methyl-CpG-binding domain protein 5	2q23.1	DNA methylation and chromatin remodeling.	7	AD	* 611472
MECP2	Methyl-CpG binding protein 2	Xq28	Transcriptional regulation through CpG island methylation, neuronal maturation, modulator of activity-dependent BDNF gene.	6; 7	XLD/XLR	* 300005
MED12	Mediator complex subunit 12	Xq13.1	Transciptional repression of neuron-specific genes in non-neuronal cell lines.	7	XLR	* 300188

MED13L	Mediator complex subunit 13-like	12q24.21	Transcriptional coactivator involved in embryogenesis, especially in brain and heart development, as part of the THRAP2 (thyroid hormone receptor associated protein 2) complex.	6; 7	AD	* 608771
MED23	Mediator complex subunit 23	6q23.2	Cofactor in transcriptional activation, role in brain development	6; 7	AR	* 605042
MID2	Midline 2	Xq22	E3 ubiquitin ligase involved in microtubule stabilization.	2	XLR	* 300204
MTOR	Mechanistic target of rapamycin	1p36.22	Protein kinase belonging to TOR complex-1 (TORC1), involved in regulation of cell growth and proliferation and mRNA translation, and TOR complex-2 (TORC2), involved in cell cycle regulation, actin cytoskeletal rearrangement, and cell survival. Role in synaptic plasticity and cancer.Association with Smith-Kingsmore syndrome (OMIM # 616638).	1; 2; 6; 7	AD	* 601231
NAA10	N(alpha)-acetyltransferase 10, NatA catalytic subunit	Xq28	Post-translational alpha acetylation of proteins and peptides, neuronal differentiation.	6; 7	XLD/XLR	* 300013
NAA15	N(alpha)-acetyltransferase 15	4q31.1	Posttranslational protein modification by tethering the NatA N-acetyltransferase complex to the ribosome. Role in neuron generation and differentiation.	6; 7	AD	* 608000
NALCN	Sodium-leak channel, nonselective	13q32.3-q33.1	Voltage-independent Na+, K+, and Ca++ channel responsible for the neuronal background sodium leak conductance --> control of neuronal excitability	4; 6	AD	* 600853
NDN	Necdin	15q11.2	Transcriptional regulation, neural differentiation and brain development. Association with Prader-Willi syndrome (OMIM # 176270).	6; 7	AD	* 602117
NDST1	N-deacetylase-sulfotransferase 1	5q33.1	Heparan sulphate biosynthesis	5	AR	* 600853
NEXMIF	Neurite extension and migration factor	Xq13.2	Mainly expressed in the cerebral cortex and cerebellum. Role in neuron development and brain function. Involvement in neurite outgrowth by regulating cell-cell adhesion via the N-cadherin signaling pathway. Regulation of gene expession.	6; 7; 8	XLD	* 300524

NFIX	Nuclear factor I/X	19p13.13	Role in hippocampal-dependent behavior, early B lymphopoiesis and myelopoiesis, neural stem/progenitor cell fate, and cell proliferation, migration, and gene expression in the subventricular zone. Association with Sotos syndrome (OMIM # 614753) and Marshall-Smith syndrome (OMIM # 602535).	1; 6; 7	AD	* 164005
NIPA1	NIPA magnesium transporter 1	15q11.2	Membrane transporter or receptor. Inhibition of BMP signaling (important for distal axonal function).	6; 8	AD	* 608145
NIPBL	NIPBL cohesin loading factor	5p13.2	Sister chromatids cohesion through loading of the cohesin complex. Loop extrusion during genome organization.	3	AD	* 608667
NLGN1	Neuroligin 1	3q26.31	Ca++-dependent postsynaptic transmembrane protein involved in synaptogenesis, synaptic plasticity and excitatory-inhibitory balanace by interacting with presynaptic neurexins with an interactive specificity according to thesplicing status of both.	6	AD	* 600568
NLGN3	Neuroligin 3	Xq13.1	Astrocyte morphogenesis and synaptogenesis.	6	XL	* 300336
NLGN4X	Neuroligin 4, X-linked	Xp22.33	Role in synaptogenesis. Association with autism and Asperger syndrome (OMIM # 300497).	6	XL	* 300427
NRXN1	Neurexin 1	2p16.3	Neuroligin-binding cell-surface receptor predominantly expressed in brain and forming a Ca^{2+}-dependent neurexin/neuroligin complex at synapses in the central nervous system for efficient neurotransmission and formation of synaptic contacts.	6	AD/AR	* 600565
NSDHL	NAD(P)H steroid dehydrogenase-like protein	Xq28	C4 demethylase involved in postsqualene cholesterol biosynthesis. Association with CHILD syndrome (OMIM # 308050) and CK syndrome (OMIM # 300831).	5	XLD/XLR	* 300275
NSUN2	NOP2/SUN RNA methyltransferase family member 2	5p15.31	Maturation of cytoplasmic tRNAs and protein translation.	7	AR	* 610916

NTM	Neurotrimin	11q25	Part of the IgLON family of GPI-anchored cell adhesion molecules (belonging to the immunoglobulin domain-containing superfamily). Neural adhesion molecule with a role in developing projection systems.	6; 8	N.A.	* 607938
OMG	Oligodendrocyte myelin glycoprotein	17q11.2	Gene embedded within an intron of the NF1 (neurofibromin 1) with growth suppressive effects. Downregulation of mitogenic signaling pathways, inhibition of neurite outgrowth, possible role in disorders of cell proliferation, such as neurofibromatosis type I.	1; 6; 8	AD	* 164345
OPHN1	Oligophrenin 1	Xq12	Rho GTPase-activating protein. Role in activity-dependent maturation and plasticity of excitatory synapses by controlling their structural and functional stability (selective enhancement of AMPA-receptor-mediated synaptic transmission, spine size control by stabilizing synaptic AMPA receptors). Interaction with proteins involved in chlathrin-mediated endocytosis	6; 8	XLR	* 300127
PAK3	p21 protein (Cdc42/Rac)-activated kinase 3	Xq23	Critical regulator of signal transduction pathways by linking Rho-GTPases with actin cytoskeleton and Map kinase cascades. Coordination of neuronal complexity, synaptic properties and dendrite/axon growth	6; 8	XLR	* 300142
PCDH19	Protocadherin 19	Xq22.1	Calcium-dependent cell adhesion molecule involved in cortex and hippocampus development likely through adhesion-based self-organization.	6; 8	XL	* 300460
PHF6	PHD finger protein 6	Xq26.3	Nucleolar protein with a likely role in transcription. Association with Borjeson-Forssman-Lehmann syndrome (OMIM # 301900).	7	XLR	* 300414
PHF8	PHD finger protein 8	Xp11.22	Fe(II) and 2-oxoglutarate-dependent N-epsilon-methyl lysine demethylase acting on histone substrates. Role in brain and craniofacial development. Function dependent on oxygen availability (possible link between maternal hypoxia and facial clefting).	6; 7	XLR	* 300560
PHIP	Pleckstrin homology domain interacting protein	6q14.1	Insulin signaling modulation, pancreatic beta cell growth and survivavalby binding to the insulin receptor substrate-1 (IRS-1). Role in substrate-specific proteolysis in a ubiquitin ligase pathway.	8	AD	* 612870
PIGN	Phosphatidylinositol glycan anchor biosynthesis class N	18q21.33	GPI-anchor biosynthesis, protein trafficking from the site of synthesis to the cell membrane, suppression of chromosomal instability.	3; 8; 9	AR	* 606097

PIGY	Phosphatidylinositol glycan anchor biosynthesis class Y protein	4q22.1	Biosynthesis of GPI, an important membrane anchor for many cellular proteins. Association with hyperphosphatasia with mental retardation syndrome 6 (OMIM # 616809).	8	AR	* 610662
PNPLA4	Patatin-like phospholipase domain containing 4	Xp22.31	Mitochondrial triacylglycerol lipase and transacetylase involved in mitochondrial oxidative phosphorylation. Highly expressed in brain and skeletal muscle.	5	XLR	* 300102
POGZ	Pogo transposable element with ZNF domain	11q21.3	Mitotic progression through dissociation of the heterochromatin-forming HP1-alpha from chromosome arms and activation of Aurora kinase B.	1	AD	* 614787
PQBP1	Polyglutamine-binding protein 1	Xp11.23	Nuclear polyglutamine-binding protein, negative regulation of transcription through binding to RNA. Association with Renpenning syndrome (OMIM # 309500).	7	XLR	* 300463
PRRT2	Proline-rich transmembrane protein 2	16p11.2	Axonal protein interacting with SNAP-25 and t-SNARE.	6	AD	* 614386
PRSS12	Serine protease 12	4q26	Extracellular multidomain serine protease associated with neural development and plasticity.	6	AR	* 606709
PTCHD1	Patched domain-containing protein 1	Xp22.11	Role in the hedgehog signaling pathway. Association with autism susceptibility.	8	XLR	* 300828
PTEN	Phosphatase and tensin homolog	10q23.31	Tumor suppressor dual-specificity phosphatase involved in negative regulation of PI3K and MAPK pathways. Possible role in inhibition of cell adhesion and migration, regulation of phosphatidylinositol signaling pathways, cell cycle and apoptosis.	1; 8	AD/AR	* 601728
PTPN11	Protein tyrosine phosphatase nonreceptor-type 11	12q24.13	Regulation of cellular response to extracellular signals by regulating the phosphotyrosine content of specific intracellular proteins.Association with LEOPARD syndrome (OMIM # 151100), Noonan syndrome (OMIM # 163950) and metachondromatosis (OMIM # 156250).	1	AD	* 176876
RAB38B	Member RAS oncogene family	Xq28	GTPase involved in the regulation of vesicular trafficking between membrane compartments, neuronal development and neurite extension, alpha-synuclein homeostasis.	6;9	XLR	* 300774

RAD21	RAD21 cohesin complex component	8q24.11	Part of a cohesin complex involved in sister chromatids cohesion during cell division. Role in transcriptional insulation. Association with Mungan syndrome (OMIM # 611376) and Cornelia De Lange syndrome (OMIM # 614701).	3; 7	AD/AR	* 606462
RAI1	Retinoic acid-induced 1	17p11.2	Positive transcriptional regulator of CLOCK gene, regulation of cyrcadian rythm.	7	AD	* 607642
RBFOX1	RNA-binding FOX1 homolog 1	16p13.3	Positive or negative regulation of tissue-specific splicing by binding to the element (U)GCAUG in mRNA precursors. Role in synaptic structure and function.	6; 7	N.A.	* 605104
RBM19	RNA-binding motif protein 19	12q24.13-q24.21	RNA-binding protein during ribosome biogenesis.	7	N.A.	* 616444
RELN	Reelin	7q22.1	Secreted glycoprotein produced by specific cell types within the developing brain. Role in activation of signaling pathways in postmitotic migrating neurons, proper positioning of neurons and neuronal layering during brain development, microtubule function in neurons. Possible role as a barrier to neuronal migration.	2; 6; 8	AD/AR	* 600514
RPS6KA3	Ribosomal protein S6 kinase A3	Xp22.2-p22.1	Growth factor-regulated serine/threonine kinase with a role in cell cycle progression, differentiation, and cell survival. Prominent expression in developing neural and sensory tissues. Association with Coffin-Lowrie syndrome (OMIM # 303600).	1; 8	XLD	* 300075
SALL1	SAL-like 1	16q12.1	Transcription regulation, regulation of higher-order chromatin structures. Regulation of ciliary function during embryogenesis.	7	AD	* 602218
SATB2	Special AT-rich sequence-binding protein 2	2q33.1	Nuclear matrix DNA-binding protein specifically binding to genomic nuclear matrix attachment regions. Role in transcriptional regulation and chromatin remodeling. Association with Glass syndrome (OMIM# 612313).	7	AD	* 608148
SCN1A	Sodium voltage-gated channel alpha subunit 1	2q24.3	Subunit of voltage-sensitive sodium channel (essential for the expression of a functional channel), action potential generation.	4; 6	AD	* 182389
SCN2A	Voltage-gated sodium channel alpha subunit 2	2q24.3	Action potential initiation and conduction, neuronal plasticity, especially during CNS development.	4; 6	AD	* 182390

SCN3A	Sodium voltage-gated channel alpha subunit 3	2q24.3	Subunit of voltage-sensitive sodium channel involved in action potential generation.	4; 6	AD	* 182391
SCN8A	Sodium voltage-gated channel alpha subunit 8	12q13.13	Voltage-dependent sodium channel involved in action potential generation and synaptic plasticity.	4; 6	AD	* 182389
SCN9A	Sodium voltage-gated channel alpha subunit 9	2q24.3	Voltage-gated sodium channel enriched in nociceptive and sympathetic neurons of the peripheral nervous system and in subcortical structures. Association with primary erythermalgia (OMIM # 133020), congenital insensitivity to pain (OMIM # 243000), seizures and Dravet syndrome (OMIM # 607208).	4; 6	AD/AR	* 603415
SETBP1	SET-binding protein 1	12q12.3	Promotion of the oncoprotein SEB (involved in cell apoptosis, cell cycle regulation, negative regulation of neuronal differentiation). Association with Schinzel-Giedion midface retraction syndrome (OMIM # 269150).	1; 6	AD	* 611060
SETD1B	SET domain-containing protein 1B	12q24.31	Domain of a histone methyltransferase complex involved in H3K4 methylation and epigenetic regulation of transcription.	7	AD	* 611055
SETD5	SET domain containing 5	3p25.3	Putative methyltransferase mainly expressed in brain and spinal cord	7	AD	* 615743
SHANK2	SH3 and multiple ankyrin repeat domains 2	11q13.3-q13.4	Neuronal development through binding to SH3 domain of contactin, cytoskeletal reorganization during neurite outgrowth, synaptic scaffolding.	2; 6	AD	* 603290
SHANK3	SH3 and multiple ankyrin repeat domains	22q13.33	Enriched at excitatory synapses. Role in receptor tyrosine kinase signaling, integration of neurotransmitter receptors into the cortical cytoskeleton at postsynaptic densities. Interaction with hyperpolarization-activated cyclic nucleotide-gated cation channel. Role in tubulogenesis, neuronal morphology and synaptic connectivity.	6; 8	AD	* 606230
SHH	Sonic hedgehog	7q36.3	Notochord patterning, regulation of polarity and morphology during embryogenesis, regulation of primitive hematopoietic cells via mechanisms dependent on downstream bone morphogenic protein signals.	2	AD	* 600725

SHROOM4	SHROOM Family member 4	Xp11.2	Role in neurulation, actin remodeling, ion channel function, and cytoskeletal architecture by modulating the spatial distribution of myosin II. Association with Stocco dos Santos X-linked mental retardation syndrome (OMIM # 300434).	2	XL	* 300579
SIN3A	SIN3 transcription regulator family member A	15q24.2	Scaffold protein for the assembly of a histone deacetylase corepressor complex together with HDACs	7	AD	* 607776
SIN3B	SIN3 transcription regulator family member 3B	19p13.11	Transcriptional repressor	7	AD	* 607777
SLC16A2	Solute carrier family 16 (monocarboxylic acid transporter) member 2	Xq13.2	Transport of thyroid hormones or their inactive metabolites into and out of the cerebrospinal fluid. Association with Allan-Herndon-Dudley syndrome (OMIM # 300523).	5	XL	* 300095
SLC2A1	Solute carrier family 2, member 1	1p34.2	Transport of glucose and dehydroascorbic acid (vitamin C). Key glucose transporter at the blood brain barrier.	5	AD	* 138140
SLC6A8	Solute carier family 6 (neurotransmitter transporter, creatine), member 8	Xq28	Temporal and spatial maintenance of the energy supply to skeletal and cardiac muscle functioning as a sodium-dependent creatine transporter	5	XLR	* 300036
SLC9A6	Solute carrier family 9, member A6	Xq26.3	Monovalent sodium-selective sodium/hydrogen exchanger (NHE) found in the membranes of intracellular organelles such as mitochondria and endosomes. Control of intracellular pH, maintenance of cellular volume, and reabsorption of sodium across renal, intestinal, and other epithelia. Most abundant in mitochondrion-rich tissues such as brain, skeletal muscle, and heart.	4	XL	* 300231
SMARCC2	SWI/SNF related, matrix associated, actin dependent regulator of chromatin subfamily c member 2	12q13.2	Repression of neurogenesis ancd control of cortical size through chromatin remodeling in a specific time window.	7	AD	* 611055
SMC1A	Structural maintenance of chromosomes 1A	Xp11.22	Chromosome cohesion, condensation and recombination, generation of cell type-specific DNA loops linked to the gene expression program of each cell. Possible role in forebrain patterning. Association with Cornelia de Lange syndrome (OMIM # 300590).	3; 7	XLD	* 300040

SMC3	Structural maintenance of chromosomes 3	10q25.2	Subunit of the cohesin complex involved in sister chromatid cohesion, DNA repair mechanisms and maintenance of genome stability.	3	AD	* 606062
SMS	Spermine synthase	Xp22.1	Synthesis of polyamines from arginine and methionine. Association with X-lined Snyder-Robinson type mental retardation (OMIM # 309583)	5	XLR	* 300105
SOX2	SRY-box 2	3q26.33	Transcriptional regulation by binding to DNA and causing its bending, inhibition of neuronal differentiation. Association with syndromic microphtalmia and optic nerve hypoplasia (OMIM # 206900).	6; 7	AD	* 184429
SPTAN1	Spectrin, alpha, non-erythrocytic 1	9q34.11	Filamentous cytoskeletal protein, heterodimer of a constant alpha-chain and variable, tissue-specific beta-chains. Regulation of receptor binding, cell cycle (inhibition of cell proliferation), actin crosslinking. Association with West syndrome.	1; 2	AD	* 182810
STAG1	Stromal antigen 1	3q22.3	Subunit of the cohesin complex involved in the control of chromosome segregation during cell division, gene transcription, DNA repair and replication.	1; 3; 7	AD	* 604358
STXBP1	Syntaxin-binding protein 1	9q34.11	Regulation of synaptic vesicle docking and fusion. Association with epileptic encephalopathy (OMIM # 612164).	6; 9	AD	* 602926
SUPT16H	SPT16 homolog, facilitates chromatin remodeling subunit	14q11.2	Component of the FACT (facilitates chromatin transcription) complex. Role in transcription elongation, DNA replication and repair.	1; 3; 7	AD	* 605012
SYN1	Synapsin I	Xp11.3-p11.2	Neuronal phosphoprotein associated with the membranes of small synaptic vesicles, role in synaptic neurotransmission, neuronal development, synaptogenesis, maintenance of mature synapses, and plasticity.	6	XLD/XLR	* 313440
SYNGAP1	Synaptic RAS-GTPase-activating protein 1	6p21.32	Brain-specific synaptic Ras GTP-ase activating protein localized to dendritic spines in neocortical pyramidal neurons, role in suppression of signaling pathways linked to NMDA receptor-mediated synaptic plasticity and AMPA receptor membrane insertion.	6; 8	AD	* 603384
SYP	Synaptophysin	Xp11.23-p11.22	The most abundant synaptic vesicle integral membrane protein by mass (10% of the total). Role in regulation of synaptic vesicle endocytosis.	6; 9	XLR	* 313475

SZT2	Subunit of KICSTOR complex	1p34.2	Protein with a superoxide dismutase motif and a peroxisomal targeting signal, mainly expressed in brain and spinal cord. Scaffold molecule forming the SZT2-GATOR1-GATOR2 complex for GATOR-dependent nutrient sensing and control of MTORC1 signaling.	5; 8	AR	* 615463
TAF1	TAF1 RNA polymerase II; TATA box-binding protein-associated factor	Xq13	Binding protein complex required for RNA polymerase II-mediated transcription of many genes.Association with X-linked dystonia-parkinsonism (OMIM # 314250).	7	XLR	* 313650
TAF2	TATA-box binding protein- associated factor 2	8q24.12	General transcription factor for RNA polymerases.	7	AR	* 604912
TBC1D24	TBC1 domain family member 24	16p13.3	Member of the Tre2-Bub2-Cdc16 (TBC) domain-containing RAB-specific GTPase-activating proteins.<br>Coordination of Rab proteins and other GTPases for the proper transport of intracellular vesicles, promotion of neurite growth and arborization, brain development, vesicle trafficking at synapses. Specific function in the stereocilia of the inner ear.	6; 9	AD/AR	* 613577
TBL1XR1	Transducin-beta-like 1 receptor 1	3q26.32	Nuclear protein interacting with histones H2B and H4 with a role in transcription mediated by nuclear receptors. Association with Pierpoint syndrome (OMIM # 602342).	7	AD	* 608628
TBX1	T-box region	22q11.21	Transcription factor, regulation of developmental processes. Association with Digeorge syndrome (OMIM # 188400), velocardiofacial syndrome (OMIM #192430), conotruncal anomaly face syndrome (OMIM # 217095), and tetralogy of Fallot (OMIM # 187500).	7	AD	* 602054
TCF4	Transcription factor 4	18q21.2	Transcription factor.	7	AD	* 602272
TECR	Trans-2,3-enoyl-CoA reductase	19p13.12	Role in the final step of microsomal long and very long fatty acid elongation.	5	AR	* 610057
TMEM5	Transmembrane protein 5	12q14.2	Glycosyltransferase involved in dystroglycan glycosylation	5	AR	* 605862
TPO	Thyroid peroxidase	2p25.3	Heme-containing protein located on the apical membranes of the thyroid follicular cell. Key role in thyroid hormone synthesis. Association with thyroid dyshormonogenesis-2A (OMIM # 274500).	5	AR	* 606765

TRAPPC9	Trafficking proteinparticle complex subunit 9	8q24.3	Enhancer of neuronal NF-κ-B signaling pathway through binding to NIK and IKK-β, downstream component of NGF in neuronal cells.	8	AR	* 611966
TRIO	Triple functional domain	5p15.2	Serine/threonine kinase guanine nucleotide exchange factor (GEF) for the family of Rho-like GTPases. Role in controlling cell proliferation.	1; 8	AD	* 601893
TRIP12	Thyroid hormone receptor interactor 12	2q36.3	Thyroid hormone receptor-interacting protein and HECT-type E3 ubiquitin-protein ligase, ubiquitination and protein labeling for proteasomal degradation, ubiquitin fusion degradation pathway, regulation of DNA damage-induced chromatin ubiquitination.	3; 8	AD	* 604506
TSC2	Tuberous sclerosis 2	16p13.13	Tumor suppressor by negatively regulating mTORC1 signaling. Role in microtubule-mediated protein transport and regulation of cellular energy response.	5; 8	AD	* 191092
TSPAN7	Tetraspanin 7	Xp11.4	Contribution in molecular complexes including beta-1 integrins. Possible role in the control of neurite outgrowth.	2; 6	XLR	* 300096
TUSC3	Tumor suppressor candidate 3	8p22	Plasma membrane magnesium transport system. Role in embryonic development.	4	AR	* 601385
UBE2A	Ubiquitin-conjugating enzyme E2A	Xq24	E2 ubiquitin-conjugating enzyme involved in the ubiquitin proteasome pathway of protein degradation. Role in DNA repair, fertility, and memory formation. Association with Nascimento-type X-linked syndromic mental retardation (OMIM # 300860).	3; 8	XLR	* 312180
UBE3A	Ubiquitin-protein ligase E3A	15q11.2	E3 ligase in the ubiquitin-proteasome pathway to degrade misfolded proteins. Role in transcriptional transactivation, dendritic patterning, synapse formation and organization, suppression of neuronal excitability via ubiquitin-mediated degradation of calcium- and voltage-gated big potassium channels (BK). Association with Angelman syndrome (OMIM # 105830).	6; 7	AD	* 601623
UBE3B	Ubiquitin-protein ligase E3B	12q24.11	E3 ligase in the ubiquitin-proteasome pathway.	8	AR	* 608047

UPF3B	UPF3B rugulator of nonsense-mediated mRNA decay	Xq25-q26	Regulation of translation through degradation of transcripts with premature termination codons.	7	XLR	* 300298
USP9X	Ubiquitin specific peptidase 9, X-linked	Xp11.4	Deubiquitination. Role in tissue-specific ciliary signal transduction pathways, neuronal growth and migration, regulation of dynamic protein-protein interaction and chromosome segregation independently of protein degradation.	6; 8	XLD/XLR	* 300072
VLDLR	Very low density lipoprotein receptor	9p24.2	Role in regulation of microtubule function in neurons and in neuronal migration during nervous system development. Association with cerebellar hypoplasia and mental retardation with or without quadrupedal locomotion 1 (OMIM # 224050).	2; 6	AR	* 192977
VPS13B	Vacuolar protein sorting 13	8q22.2	Transmembrane protein involved in Golgi complex scaffolding and trafficking, vesicle-mediated transport and sorting of proteins within the cell and in the development and function of the eye, hematological system, and CNS (neurite outgrowth). Association with Cohen syndrome (OMIM # 216550).	2; 9	AR	* 607817
ZBTB18	Zinc finger- and BTB domain-containing protein 18	1q44	Chromatin assembly and transcriptional repression of key proneurogenic genes.	6; 7	AD	* 608433
ZBTB20	Zinc finger- and BTB domain-containing protein 20	3q13.31	Member of the POK (POZ and Kruppel) family of transcriptional repressors. Expression detected in early hippocampal neurons, cerebellar granule cells, and gliogenic progenitors, as well as in differentiated glia. Possible role in hematopoiesis, oncogenesis, and immune responses. Association with Primrose syndrome (OMIM # 259050).	6; 7	AD	* 606025
ZDHHC9	Zinc finger DHCC domain-containing protein 9	Xq26.1	Protein acyltransferase activity on HRAS and NRAS. Association with Raymond type X-linked syndromic mental retardation (OMIM # 300799).	8	XL	* 300646
ZEB2	Zinc finger E box-binding homeobox 2	2q22.3	DNA-binding transcriptional repressor interacting with activated SMADs, the transducers of TGF-beta signaling, and with the nucleosome remodeling and histone deacetylation (NURD) complex. Association with Mowat-Wilson syndrome (OMIM # 235730).	7	AD	* 605802
ZMYM2	Zinc finger MYM-type- containing 2	13q12.11	Transcriptional regulation.	7	AD	* 602221

ZNF41	Zinc finger protein 41	Xp11.3	Transcriptional repression, chromatin remodeling.	7	XL	* 314995
ZNF674	Zinc finger protein 674	Xp11	Regulation of transcription by forming a nuclear receptor corepressor complex. Crucial role in human cognitive funtioning.	6; 7	XL	* 300573
ZNF711	Zinc finger protein 711	Xq21.1-q21.3	Transcriptional regulation in brain development through bindong to the promoter of target genes and recruitment of PHF8 histone demethylase.	7	XL	* 314990
ZNF81	Zinc finger protein 81	Xp11.23	Krueppel-type zinc finger gene (with a Krueppel repressor domain). RNA polymerase II-specific transcription factor. Role in gene silencing.	7	XL	* 314998

*1. Cell cycle regulation; 2. Cell structure and polarity; 3. Genomic stability/DNA repair; 4. Membrane polarity/electrochemical gradient; 5. Metabolic pathways; 6. Brain function/development and neuronal signaling; 7. Transcriptional/translational regulation and cell differentiation; 8. Intra- and intercellular signal transduction; 9. Vesicular trafficking.

Abbreviations: AD, autosomal dominant; AR, autosomal recessive; N.A., not applicable; XL, X-linked; XLD, X-linked dominant; XLR, X-linked recessive.
