# Supplementary material for: The Usefulness of a Targeted Next Generation Sequencing Gene Panel in Providing Molecular Diagnosis to Patients With a Broad Spectrum of Neurodevelopmental Disorders
Source: Front Genet. 2022 Aug 11;13:875182. doi: 10.3389/fgene.2022.875182 (PMC9403311; doi:10.3389/fgene.2022.875182)
Supplement: Supplementary file 3 [file Table2.pdf]

Supplementary Table 2 **Causative variants detected in the 71 patients.**

Patient code	Sex	Gene	Variant	Mode of inheritance	Variant segregation	Variant Classification	ID	ASD	Epilepsy	Phenotype correlation
**9916**	M	***AKT3***	NM_005465.7:c.503A>G p.Lys168Arg	AD	Mat	P	x	-	-	Yes
**8513**	F	***ANKRD11***	NM_013275.6:c.2408-2412del p.Lys803ArgfsTer5	AD	*de novo*	P	x	-	-	Yes
**10231**	F	***ANKRD11***	NM_013275.6:c.1903_1907del p.Lys635GlnfsTer26	AD	Mat	P	x	-	x	Yes
**10462**	M	***ANKRD11***	NM_013275.6:c.4384dupA p.Arg1462LysfsTer92	AD	*de novo*	P	x	-	x	Yes
**88-19**	F	***ARID1B***	NM_017519.3:c.5003_5004insC p.Gln1668HisfsTer38	AD	*de novo*	P	-	-	x	Yes
**212-20**	M	***ARID1B***	NM_017519.3:c.4258_4259insC p.Gly1420AlafsTer109	AD	*de novo*	P	-	-	x	Yes
**482-19**	M	***ATP1A2***	NM_000702.4:c.1091C>T p.Thr364Met	AD	Mat	P	-	-	x	Yes
**78-20**	F	***ATP1A2***	NM_000702.4:c.2750C>T p.Thr917Met	AD	*de novo*	P	-	-	x	Yes
**514-21**	F	***ATP1A2***	NM_000702.4:c.904del p.Val302SerfsTer25	AD	nd	P	x	-	-	Yes

**10727**	M	***CACNA1A***	NM_000068.4: c.1067T>C<br><br>p.Leu356Pro	AD	*de novo*	P	x	-	-	Yes
**927-19**	F	***CACNA1A***	NM_000068.4: c.5005C>T<br>p.Arg1669Ter	AD	*de novo*	P	x	-	x	Yes
**8804**	M	***CACNA1A***	NM_000068.4: c.3958G>A<br>p.Asp1320Asn	AD	*de novo*	P	x	-	x	Yes
**928-19**	F	***CASK***	NM_003688.3:c.252_255del<br>p.Asp84GlufsTer20	X-linked	*de novo*	P	x	-	x	Yes
**10635**	F	***CASK***	NM_003688.3:c.1090A>G<br>p.Ser364Gly	X-linked	nd	LP	x	-	-	Yes
**10851**	M	***CDKL5***	NM_003159.2:c.2798-1G>A	X-linked	Mat	P	-	x	-	Yes
**513-20**	F	***CDKL5***	NM_003159.2:c.484G>T<br>p.Glu162Ter	X-linked	*de novo*	P	x	x	x	Yes
**8325**	F	***CHD7***	NM_017780.4:c.5665+1G>C	AD	nd	P	x	-	-	Yes
**10458**	M	***CREBBP***	NM_004380.3: c.3832G>A<br>p.Glu1278Lys	AD	*de novo*	P	x	-	-	Yes
**948-19**	M	***CTNNB1***	NM_001904.4:c.936_936+1insTG<br>p.Leu313CysfsTer13	AD	*de novo*	P	x	-	-	Yes
**8511**	M	***CTNND2***	NM_001332.4: c.313C>T<br>p.Gln105Ter	AD	Mat	P	-	-	-	Partial

**9481**	F	***DDX3X***	NM_001356.5:c.1171-1G>T	X-linked	*de novo*	P	x	-	-	Yes
**8836**	M	***DHCR7***	NM_001360.3:c.964-1G>C<br>NM_001360.3:c.523G>A p.Asp175Asn	AR<br>AR	Mat<br>Pat	P<br>P	x	-	-	Yes
**765-20**	M	***EHMT1***	NM_024757.5:c.523C>G p.Thr1133Arg	AD	*de novo*	P	x	x	-	Yes
**345-19**	F	***FOXG1***	NM_005249.5: c.694A>G p.Asn232Asp	AD	*de novo*	P	x	-	x	Yes
**213-20**	M	***FOXP1***	NM_032682.6:c.570-1G>C	AD	*de novo*	P	x	-	x	Yes
**749-20**	F	***FOXP1***	NM_032682.6:c.1240dup p.Leu414ProfsTer47	AD	*de novo*	P	x	-	-	Yes
**574-19**	F	***FOXP1***	NM_032682.6:c.777_780del p.Lys259AsnfsTer6	AD	*de novo*	P	x	x	x	Yes
**684-19**	F	***GABRA1***	NM_000806.5:c.641G>A p.Arg214His	AD	*de novo*	P	-	-	x	Yes
**9226**	F	***GATAD2B***	NM_020699.4:c.332G>A p.Arg111Gln	AD	*de novo*	P	x	-	-	Yes
**490-19**	F	***GDI1***	NM_001493.3:c.1036C>T p.Gln346Ter	X-linked	Pat	P	x	-	-	Partial

**2-20**	M	***GNAS***	NM_080425.3:c.303_304insT p.Met102TyrfsTer6	AD	nd	P	x	-	-	Yes
**446-20**	F	***GNAS***	NM_080425.3:c.214G>A p.Gly716Ser	AD	nd	LP	-	x	-	Yes
**796-19**	F	***GNAS***	NM_080425.3:c.2936G>A p.Arg979Gln	AD	Mat	P	x	-	-	Yes
**328-19**	M	***GRIN2B***	NM_000834.5: c.1385T>C p.Ile462Thr	AD	*de novo*	P	x	-	x	Yes
**422-20**	F	***GRIN2B***	NM_000834.5:c.286G>A p.Gly96Arg	AD	*de novo*	P	x	-	-	Yes
**947-19**	M	***KANSL1***	NM_001193466.2:c.1653-2A>G	AD	nd	P	-	-	-	Yes
**275-19**	M	***KANSL1***	NM_001193466.2:c.2021A>G p.Asp674Gly	AD	Mat	P	-	x	-	Partial
**8579**	M	***KCNQ2***	NM_172107.4:c.1090G>C p.Glu364Gln	AD	Pat	LP	x	-	x	Yes
**525-21**	M	***KCNQ2***	NM_172107.4:c.641G>A p.Arg214Gln	AD	*de novo*	P	-	-	x	Yes
**10617**	M	***KDM5B***	NM_006618.5:c.3133C>T p.Arg1045Ter	AD	Pat	P	x	-	-	Yes
**1002-19**	M	***KDM6A***	NM_021140.3:c.1952C>A p.Ser651Ter	X-linked	Mat	P	x	-	-	Yes

**32-19**	F	***MECP2***	NM_004992.4:c.397C>T p.Arg133Cys	X-linked	*de novo*	P	x	-	x	Yes
**257-19**	F	***MED13L***	NM_015335.4:c.4963delG p.Glu1655LysfsTer5	AD	*de novo*	P	x	-	-	Yes
**7790**	F	***NAA10***	NM_003491.4:c.384T>A p.Phe128Leu	X-linked	*de novo*	P	x	-	x	Yes
**254-19**	F	***NALCN***	NM_052867.4:c.2294G>C p.Arg765Thr	AD	Pat	LP	-	-	x	Yes
**139-20**	F	***NEXMIF***	NM_001008537.3:c.2552C>A p.Ser851Ter	X-linked	*de novo*	P	x	-	x	Yes
**9322**	M	***NLGN1***	NM_014932.5:c.1156G>T p.Gly386Ter	AD	Pat	P	x	-	-	Partial
**494-19**	F	***PAK3***	NM_001128168.3: c.500C>G p.Thr167Arg	X-linked	Mat	LP	-	-	x	Yes
**781-20**	F	***PHIP***	NM_017934.7:c.1096G>T p.Asp366Tyr	AD	*de novo*	P	x	-	-	Yes
**85-19**	F	***PRRT2***	NM_145239.3:c.649dupC p.Arg217ProfsTer8	AD	Mat	P	-	-	x	Yes
**9574**	M	***RAI1***	NM_030665.4:c.2000T>C p.Leu667Pro	AD	*de novo*	LP	x	-	-	Yes
**10488**	F	***SCN1A***	NM_001165963.4:c.2266A>T p.Lys756Ter	AD	nd	P	-	-	x	Yes

**10457**	F	***SCN1A***	NM_001165963.4: c.695-6_695-3del	AD	*de novo*	P	x	-	x	Yes
**11-19**	F	***SCN1A***	NM_001165963.4: c.4793A>T p.Tyr1598Phe	AD	Mat	LP	-	x	-	Partial
**88-20**	F	***SCN1A***	NM_006920.6:c.5104G>T p.Asp1702Tyr	AD	*de novo*	P	-	x	-	Partial
**10463**	F	***SCN8A***	NM_014191: c.4594A>T p.Ile1532Phe	AD	*de novo*	P	x	-	x	Yes
**81-19**	M	***SETD1B***	NM_001353345.2:c.5291_5292insA p.Asn1765GlnfsTer9	AD	*de novo*	P	x	-	x	Yes
**9137**	M	***SHANK2***	NM_133266.5:c.769C>T p.Arg257Ter	AD	*de novo*	P	x	-	-	Yes
**9320**	F	***SLC2A1***	NM_006516.4:c.388G>A p.Gly130Ser	AD	Mat	P	-	-	x	Yes
**483-19**	M	***SLC2A1***	NM_006516.4:c.988C>T p.Arg330Ter	AD	*de novo*	P	x	-	x	Yes
**8697**	M	***SLC2A1***	NM_006516.4:c.85_89del p.Asn29TrpfsTer58	AD	nd	P	-	-	x	Yes
**10360**	F	***SLC2A1***	NM_006516.4: c.967G>A p.Val323Met	AD	Mat	LP	-	-	x	Yes
**12-21**	F	***SLC2A1***	NM_006516.4:c.109C>T p.Gln37Ter	AD	nd	P	-	-	x	Yes

**1-19**	F	***SLC9A6***	NM_001042537.1:c.264C>G p.Ile88Met	X-linked	*de novo*	LP	x	x	-	Yes
**84-19**	F	***SMARCC2***	NM_003075.5:c.2984dupC p.Ala996CysfsTer16	AD	nd	P	x	-	x	Yes
**896-19**	F	***SYNGAP1***	NM_006772.3:c.509G>A p.Arg170Gln	AD	*de novo*	P	-	x	x	Yes
**559-19**	M	***TPO***	NM_000547.5:c.1472G>A p.Arg491His	AR	Mat/Pat	P	x	-	-	Yes
**298-19**	M	***TBX1***	NM_080646.1:c.1010-2A>G	AD	nd	P	x	-	-	Yes
		UBE3A	NM_000462.5: c.2576_2579del p.Lys859ArgfsTer4	AD	nd	P				Yes
**10456**	M	***VPS13B***	NM_017890.4:c.9406-1G>T	AR	Pat	P	x	-	-	Yes
			del ex4-7	AR	Mat	P				Yes
**211-20**	M	***ZMYM2***	NM_197968.4:c.1765C>T p.Arg589Ter	AD	Pat	P	x	x	-	Yes
**8634**	F	***ZMYM2***	NM_197968.4:c.474dup p.Ser159GlnfsTer6	AD	*de novo*	P	x	-	-	Yes

Abbreviations: AD, autosomal dominant; AR, autosomal recessive; ASD, autism spectrum disorder; F, female; ID, intellectual disability; LP, likely pathogenic; M, male; Mat, maternal; nd, not determined; P, pathogenic; Pat, paternal.
