# Supplementary figures and images for: The Usefulness of a Targeted Next Generation Sequencing Gene Panel in Providing Molecular Diagnosis to Patients With a Broad Spectrum of Neurodevelopmental Disorders
Source: Front Genet. 2022 Aug 11;13:875182. doi: 10.3389/fgene.2022.875182 (PMC9403311; doi:10.3389/fgene.2022.875182)

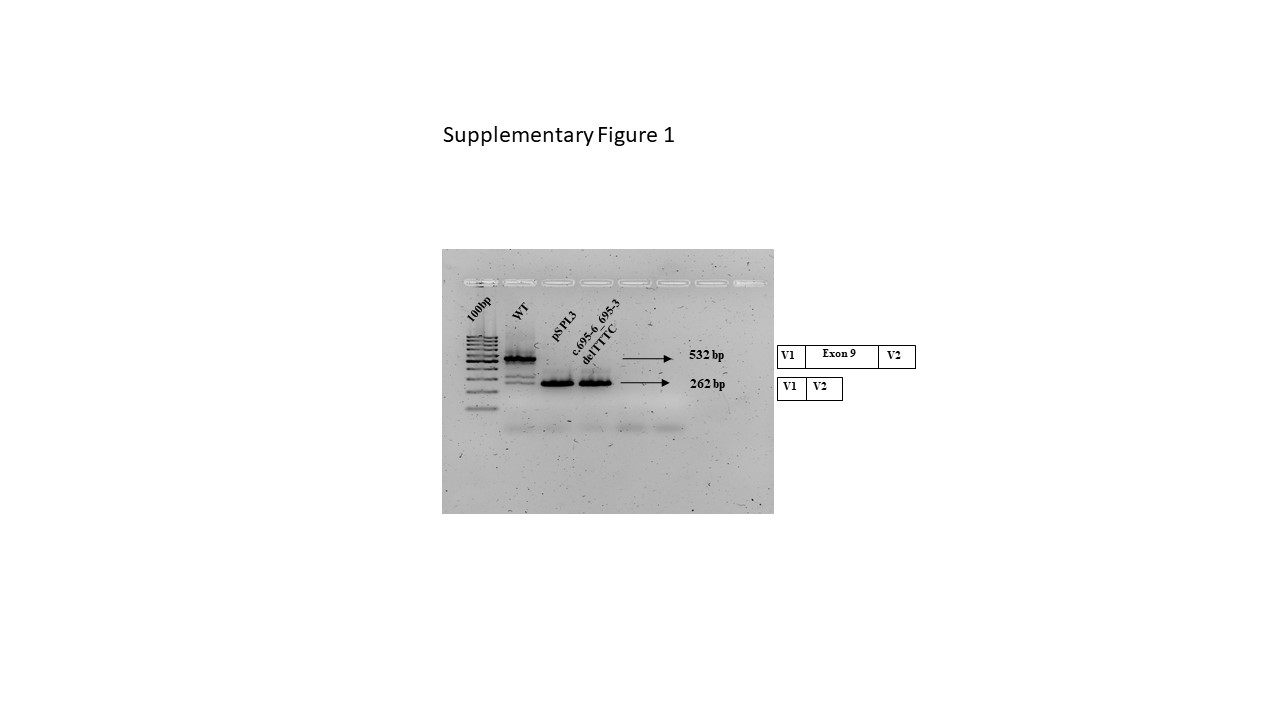

Supplement: Supplementary file 4 [file Image1.jpeg]
