# Supplementary material for: The Usefulness of a Targeted Next Generation Sequencing Gene Panel in Providing Molecular Diagnosis to Patients With a Broad Spectrum of Neurodevelopmental Disorders
Source: Front Genet. 2022 Aug 11;13:875182. doi: 10.3389/fgene.2022.875182 (PMC9403311; doi:10.3389/fgene.2022.875182)
Supplement: Supplementary file 5 [file DataSheet1.pdf]

Legend to Supplementary Figure 1

Gel electrophoresis of RT-PCR products from exon trapping assay of transfected U2O-S cells.

The wild-type (WT) construct produced a fragment of 532 bp corresponding to the correct usage of the canonical splice sites (lane 2). Spliced product from cells transfected with vector containing no inserted gDNA (empty vector) is indicated with a band size of 262 bp (lane 3). The construct bearing c.695-6_695-3delTTTC showed a band of 262 bp corresponding to an aberrant mRNA (lane 4) originated by the skipping of the 270 bases of the exon 9.

V1 and V2 represent the pSPL3 vector exons.

Primer sequences used for exon-trapping assay

**Primer**	**sequence 5' → 3'**
SCN1A_Exon9_SacI	ATATATGAGCTCCTGACCTCTGACCCTAATGAAAA
SCN1A_Exon9_BamH1	ATATATGGATCCTGGGTACATGGTAGGTGTTTC
